# Diterpenes: Nature’s Hidden Gems of Immunomodulation

**DOI:** 10.3390/ijms26052250

**Published:** 2025-03-03

**Authors:** Josiane Elizabeth Almeida, André Correa de Oliveira, Carlos Eduardo de Castro Alves, Selino Monteiro Costa Filho, Elaine Cristina Pacheco de Oliveira, Juliana Pavan Zuliani, Gemilson Soares Pontes

**Affiliations:** 1 Graduate Program in Basic and Applied Immunology, Federal University of Amazonas (UFAM), Manaus 69080-900, AM, Brazil; 2 Analytical Multidisciplinary Support Center, Federal University of Amazonas (UFAM), Manaus 69080-900, AM, Brazil; andrebiologo2011@gmail.com; 3 Laboratory of Virology and Immunology, Society, Environment and Health Coordination, National Institute of Amazonian Research (INPA), Manaus 69080-001, AM, Brazil; alveseduardo71@gmail.com; 4 Biotechnology and Medicinal Plants Laboratory, Federal University of Western Pará (UFOPA), Santarém 68040-255, PA, Brazil; selinofilho2018@gmail.com (S.M.C.F.); elaine.ibef@gmail.com (E.C.P.d.O.); 5 Laboratory of Cellular Immunology Applied to Health, Oswaldo Cruz Foundation (FIOCRUZ), Porto Velho 21040-900, RO, Brazil; juliana.zuliani@fiocruz.br; 6 Graduate Program in Hematological Sciences, University of Amazonas State (UEA), Manaus 69080-010, AM, Brazil

**Keywords:** terpenes, secondary metabolism, immunomodulatory, pharmacological potential

## Abstract

Natural products, especially specific metabolites found in many medicinal plants, exhibit extensive therapeutic potential due to their diverse biological characteristics. Among these compounds, diterpenes stand out for their active principles described in phytochemical studies. Diterpenes exhibit immunomodulatory effects by influencing the production of cytokines and other signaling molecules involved in the immune response. These actions contribute to achieving a more balanced immune profile. The ability to selectively and harmoniously modulate the immune response positions compounds derived from natural products is a promising research field in the development of immunomodulatory therapies. Due to the broad biological activities of diterpenes, the use of molecular docking emerges as a relevant tool for the quantitative screening of a large number of these substances. This review comprehensively examines the pharmacological potential of diterpenes in modulating the immune system. It highlights the existing experimental evidence supporting the efficacy and safety of these compounds as potential treatment for immune dysfunctions. Ultimately, this review aims to contribute to the development of new therapeutic strategies in this field.

## 1. Introduction

Substances synthesized by plants are recognized as secondary metabolites or natural products, classified into phenolics, terpenes, and alkaloids, with terpenes being the most abundant group in nature, with over 90,000 identified substances [1,2]. They are further categorized based on the number of carbons in the structural skeleton of the substance, classified as Hemiterpenes (C5), Monoterpenes (C10), Sesquiterpenes (C15), Diterpenes (C20), Sesterterpenes (C25), Triterpenes (C30), Tetraterpenes (C40), and Politerpenes (above C40) [3,4]. 

The significant diversity of terpenes is correlated with structural skeleton functionalization along with functional groups, explored for pharmacological purposes [5]. Secondary metabolites, especially diterpenes, are widely enriched with a broad range of pharmacological properties for the treatment of breast cancer, diabetes, rheumatoid arthritis, and lung cancer, among others [6,7]. Clinically used drugs, commercially available, such as paclitaxel (classified as a diterpenoid), oridonin, ginkgolide, or andrographolide, are derived from diterpenes [8].

Currently, diterpenes and their bioactive derivatives have shown antineoplastic and/or immunomodulatory properties [9,10]. These compounds exert their effects disrupting the cell cycle, triggering apoptotic or non-apoptotic pathways, and inhibiting angiogenesis, ultimately suppressing the proliferation and spread of cancer cells [11]. Moreover, under specific conditions, they can be used as adjuvant therapies to alleviate the side effects of chemotherapy [12]. 

Due to the increasing understanding of immune system modulation and its benefits in various diseases, the investigation of the immunomodulatory potential of natural products, particularly diterpenes and their bioactive derivatives has been gaining more prominence [13,14]. This is primarily attributed to their influence on monocytes and their pathogen recognition receptors [15]. Adapter proteins can stimulate the transcription of nuclear factor kappa B (NF-kB) in cellular activation, which induces the expression of cytokines, chemokines, antimicrobial peptides, and co-stimulatory molecules [16].

This review provides a comprehensive analysis of the diverse immunomodulatory properties of diterpenes. We delve into their structural variety, biological functions, and therapeutic potential, with a particular focus on their ability to modulate immune responses.

By exploring the mechanisms through which diterpenes employ their effects, including cell cycle disruption, angiogenesis inhibition, and immune system regulation, we highlight their promising role in advancing pharmacological research and therapeutic applications.

## 2. In Silico Screening of Diterpenes: Perspectives and Limitations

In the pursuit of predicting and identifying substances with promising biological activities, molecular docking has been successfully incorporated into research involving complex biological and chemical systems [17]. It aims to predict and identify bioactive substances by exploring the conformations of ligands within receptor binding sites and estimating the free binding energy between the ligand and receptor. This helps evaluate critical phenomena involved in the in silico intermolecular recognition process [18,19].

Several in silico studies have reported the identification of active substances and anti-inflammatory mechanisms [20], coumarins and their derivatives, lignans, phenols, alkaloids [21], amides, among others, including diterpenes with structural and non-structural proteins of DENV, ZIKV, and CHIKV [22], Alzheimer’s disease [23], AChE, and BChE [24].

Due to the broad range of biological activities exhibited by diterpenes, molecular docking is a significant tool for the quantitative screening of diterpenes [18]. For instance, the diterpenes moluccelactone and genkwanin isolated from the plant *Moluccella aucheri* Scheen (Syn. *Otostegia aucheri*) (Lamiaceae) showed anti-acetylcholinesterase activity with binding energies of −12.2 and −10.07 kcal/mol, respectively. They formed pi-pi and pi-alkyl bonds with residues Tyr337, Phe338, and Trp86 [25].

Alpha-glucosidase is an intestinal enzyme that catalyzes the final step in carbohydrate digestion, converting carbohydrates into monosaccharides [25]. Inhibition of α-glucosidase activity can effectively reduce blood sugar levels. Diterpenes such as Abeo–20 (10 → 9)−16α, 17-dihydroxy−1(10)-ent-kaur−19-oic acid, and villanovane II extracted from coffee inhibited α-glucosidase with IC_50_ values of 54.58 ± 4.2 and 149.92 ± 2.52, coupled with binding energies of −9.2 and −8.7 kcal/mol, interacting with residues GLU277, ARG315, and PHE303 [26].

The inhibition of the main protease of SARS-CoV-2 and papain-like protease was promoted by abietane-type diterpenes dihydrotanshinone, tanshinone C, tanshinone A, and tanshinone B isolated from branches of *Glyptostrobus pensilis* (Cupressaceae) with binding energies ranging from −6.6 to −8.5. The binding involved residues Thr75, Pro129, Tyr172, Lys200, Lys274, and Cys284 [27].

In conclusion, molecular docking has become an invaluable tool for predicting and identifying substances with promising biological activities within complex biological and chemical systems [23]. This accelerates the discovery and development of novel therapeutics derived from natural products like diterpenes [25].

## 3. Diversity of Diterpenes and Biological Activities

The physicochemical characteristics of diterpenes are correlated with the type of skeleton and functional groups [9]. These molecules display acidic properties if they contain carboxyl groups. Additionally, they fluoresce under ultraviolet light if they possess aromatic rings or conjugated double bonds. Furthermore, they are non-volatile and only soluble in water when bound to sugars (glycosides) [6]. Diterpenes with nitrogen atoms in the skeleton are classified as alkaloidic diterpenes, conferring them basic characteristics [28].

**Figure 1:**
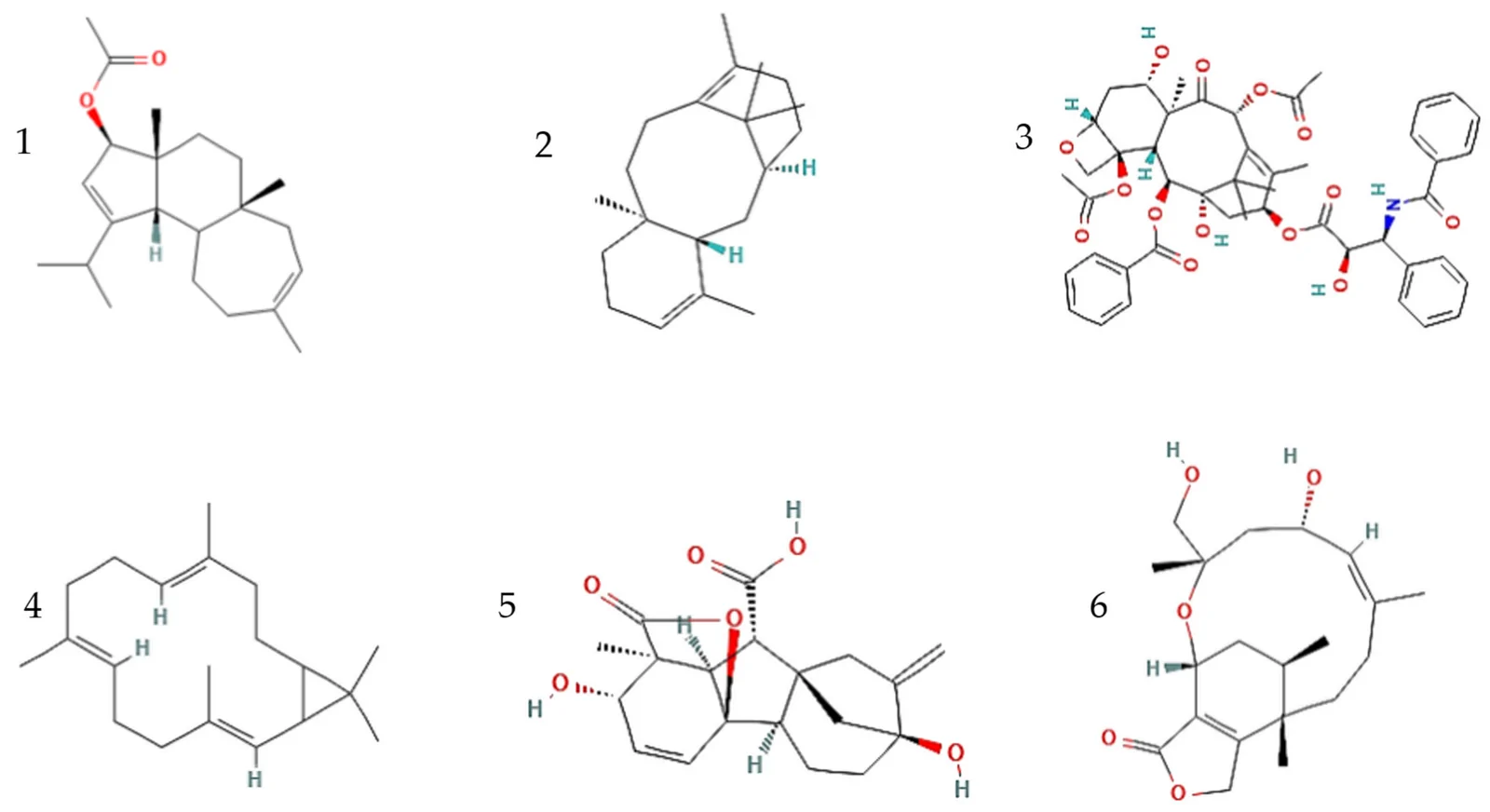
Structure of representative diterpenes. (1) acyclic diterpene, (2) monocyclic diterpene, (3) bicyclic diterpene, (4) tricyclic diterpene, (5) tetracyclic diterpenes, (6) macrocyclic diterpene.

Diterpenes are classified based on the number of rings in their structural skeleton, grouped into acyclic, monocyclic, bicyclic, tricyclic, tetracyclic, and macrocyclic structures [1] (Figure 1). Acyclic diterpenes are less frequently found in nature and have an unusual linear structure, yet they play essential roles in various biological research fields [3,7]. Examples include facmines A–C, isolated from *Aphanamixis polystachya* (Meliaceae), that exhibit inhibitory effects on nitric oxide production (IC_50_ of 6.71–15.36 μmol/L) and downregulate the nitric oxide synthase (iNOS) expression in LPS-induced RAW 264.7 macrophages [29]. Phytol, a crucial precursor for the synthesis of vitamins K1 and E, induces reactive oxygen species-mediated apoptosis by activating caspase-9 and -3 through TRAIL, FAS, and TNF receptors. Additionally, it inhibits the tumor progression factor glucose-6-phosphate dehydrogenase in the lung carcinoma cell line (A549) [30].

Monocyclic diterpenes are characterized by having a single ring in their core structure [5]. Representative compounds include sauchuchinenones A–D, afpolins A and B, isolated from *Saururus chinensis* (Lour.) Baill (Saururaceae) and A. polystachya. These monocyclic diterpenes exhibit antimicrobial activity against *Aeromonas hydrophila* ATCC^®^ 7966, *Klebsiella pneumoniae* ATCC^®^ 13883, *Acinetobacter baumannii* ATCC^®^ 19606TMA, and *Escherichia coli* ATCC 2599 (MIC > 50 mg/mL) [31,32].

Bicyclic diterpenes contain two rings in their carbon skeleton and are classified into three major subgroups: clerodanes, halimanes, and labdanes [33]. The labdane subgroup is widely studied due to its antibacterial, antimutagenic, cytotoxic, cytostatic, and anti-inflammatory activities [33,34,35]. A notable example is ent-polialtic acid (PA), isolated from the oleoresin of *Copaifera lucens*, which exhibits anticariogenic and antiparasitic activity by inhibiting the growth of cariogenic bacteria and the parasite *Toxoplasma gondii* [16]. In contrast, clerodanes are known for their antifeedant properties against insects and their anticancer and antifungal activities [36,37,38]. 

Tricyclic diterpenes, characterized by three rings in their carbon skeleton, play an essential role in the pharmaceutical industry due to their anti-inflammatory, antiviral, anticancer, antimalarial, and antimicrobial properties [39,40,41]. Representative examples include levopimaric acid, ciate, and gangerencastane acids. Additionally, tricyclic diterpenes are significant in the textile industry for dye manufacturing [1]. 

Tetracyclic diterpenes, including cauranoic acids, rebaudiosides, cafestol, and gibberellins, are biosynthesized through the cyclization of tricyclic diterpenes [42]. Gibberellins are classified based on their carbon count into C20-GAs and C19-GAs [43]. Their polarity is influenced by hydroxyl and carboxyl group content, saturation level, and the presence of methylene or sugar residues [42]. Some tetracyclic diterpenes, such as rebaudiosides, are known for their sweetening properties [44].

Macrocyclic diterpenes are complex, mostly oxygenated polycyclic structures that exhibit important biological activities, including anti-inflammatory, antitumor, antiviral, and anticancer properties [1]. Notable compounds include cembrane-type diterpenes, which act as TNF-α inhibitors [45], neocucurbins A–C and their derivatives D–G, which function as antimicrobial agents [26], and eufzicopias A–I, which inhibit the NLRP3 inflammasome [40].

In conclusion, the diversity of diterpenes is of great significance due to their broad spectrum of biological activities, making them valuable targets for pharmaceutical and medical research [6]. This presents promising opportunities for the development of novel drugs and therapies to address various health conditions [26].

### Biosynthesis of Diterpenes

**Figure 2:**
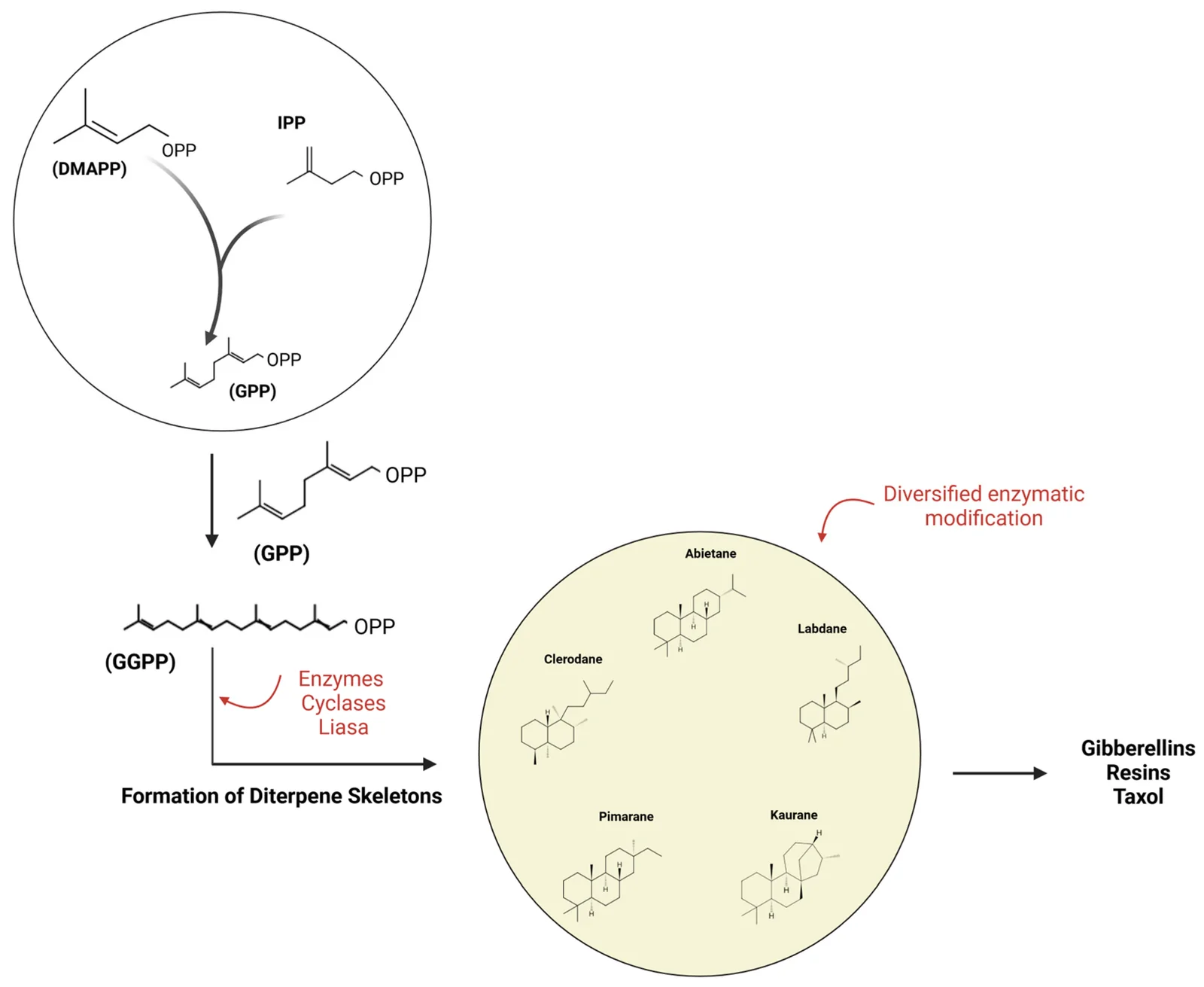
Diterpenoid biosynthesis. The synthesis begins with the formation of IPP and DMAPP, which condense to form GGPP. GGPP is then converted into different diterpene skeletons through the action of enzymes such as cyclases and lyases. Subsequently, additional enzymatic modifications, mediated by P450 monooxygenases and transferases, generate a wide diversity of bioactive compounds, including abietane, clerodane, labdane, pimarane, and kaurane.

Diterpene biosynthesis usually begins with the production of isopentenyl pyrophosphate (IPP) and dimethylallyl pyrophosphate (DMAPP) (Figure 2) [46]. These compounds serve as common intermediates in either the methylerythritol phosphate (MEP) or the mevalonate (MVA) pathway. Geranylgeranyl diphosphate (GGPP) is formed through the condensation of IPP and DMAPP [46]. Subsequently, diterpene synthase enzymes act on GGPP to produce diverse diterpene skeletons, which can undergo hydroxylation and oxidation by other enzymes to form diterpene acids. The cyclization of GGPP, mediated by enzymes, leads to the formation of carbocations and subsequent molecular rearrangement, resulting in the creation of various diterpene nuclei [47].

The diterpene biosynthesis pathway begins with the formation of geranylgeranyl pyrophosphate (GGPP), which, through enzymes such as diterpene synthases, generates various structural skeletons (Figure 2) [4]. These skeletons can be modified by additional enzymes, such as P450 monooxygenases and transferases, leading to a great diversity of bioactive compounds [47,48]. These derivatives play crucial roles in natural processes, such as defense against pathogens and herbivores, as well as possessing therapeutic potential, including in anticancer treatments. The Figure 2 illustrates this complex enzymatic modification that results in molecules with various biological and pharmacological applications.

Enzymes known as diterpene synthases catalyze the transformation of the 20-carbon GGPP into diterpenes, which are then hydroxylated and oxidized by other enzymes to produce diterpene acids [48]. The cyclization reactions of GGPP, catalyzed by enzymes, result in the formation of carbocations and subsequent molecular rearrangement, leading to the creation of a variety of diterpene nuclei [48]. The formation of these carbocation rearrangements can occur through two pathways: the first pathway involves the protonation of GGPP, resulting in combined cyclizations followed by the loss of a proton from a methyl group, leading to the formation of (-)-copalyl diphosphate. The second pathway occurs through an alternative folding, resulting in the formation of an enantiomeric product with opposite configurations at chiral centers, namely (+)-copalyl diphosphate (labdadienyl diphosphate) [49,50,51].

After an intramolecular proton transfer and a 1,2-methyl migration, deprotonation at one of the sites produces the main products levopimaradiene, abietadiene, neoabietadiene, and palustradiene. The biosynthesis of other tricyclic diterpenes can occur through deprotonation of the carbocation without rearrangements or by deprotonation of pimaradienyl carbocation intermediate products. The abietadiene synthase contains two aspartate-rich motifs, consistent with the two active sites: a DXDD motif in the N-terminal domain, indicative of a class II terpene synthase fold, and a DDXXD motif in the C-terminal domain, indicative of a class I terpene synthase fold [52].

## 4. Unraveling the Immunoregulatory Potential of Diterpenes 

Recent studies have revealed the substantial immunomodulatory properties of diterpenes, particularly highlighted in contexts such as tuberculosis infections and breast cancer [53,54,55]. This burgeoning field of research into the effects of diterpenes on innate immunity and cytokine production has ignited novel prospects for the development of more effective immunomodulatory therapies, showcasing their potential in cancer cell proliferation, gene expression regulation, differentiation, and apoptosis modulation [53,54,55,56]. 

The diterpenic acids grandiflorolic, kaurenoic, and trachylobanolic isolated from *Helianthus annuus* L. have shown anti-inflammatory properties [57]. These compounds, in non-toxic concentrations, have demonstrated the ability to reduce the production of critical inflammatory mediators such as nitric oxide (NO), prostaglandin E_2_ (PGE_2_), and TNF-α, in addition to inhibiting the expression of pro-inflammatory enzymes, including NOS-2 and COX-2. Furthermore, there was a significant reduction in ear edema in murine models, accompanied by inhibition of myeloperoxidase (MPO) activity, indicating reduced inflammatory cell infiltration. These results emphasize the therapeutic potential of *Helianthus annuus* diterpenoids as anti-inflammatory agents.

Moreover, investigations into the effects of diterpenes on humoral and cellular immune responses offer auspicious avenues for the development of immunomodulatory therapies and intervention strategies for conditions entailing immune system dysfunctions [58]. Lastly, delving into the influence of diterpenes on inflammasome pathways represents an emerging research frontier, poised with significant implications for managing inflammatory conditions and probing into new therapeutic opportunities rooted in natural compounds [59]. The growing acknowledgment of diterpenes’ immunomodulatory properties indicates their potential for therapeutic applications, thus encouraging further research in the field of immunomodulation.

### 4.1. Anti-Inflammatory Properties of Diterpenes

The biological activities of diterpenic acids, such as anti-inflammatory, antimicrobial, gastroprotective, and antitrypanosomal effects, have made them a focus of extensive scientific research [47,60,61]. Of particular note is the anti-inflammatory activity (Table 1) of these compounds, which has been widely explored due to their therapeutic potential and application in the modulation of inflammatory processes [60].

**Figure 3:**
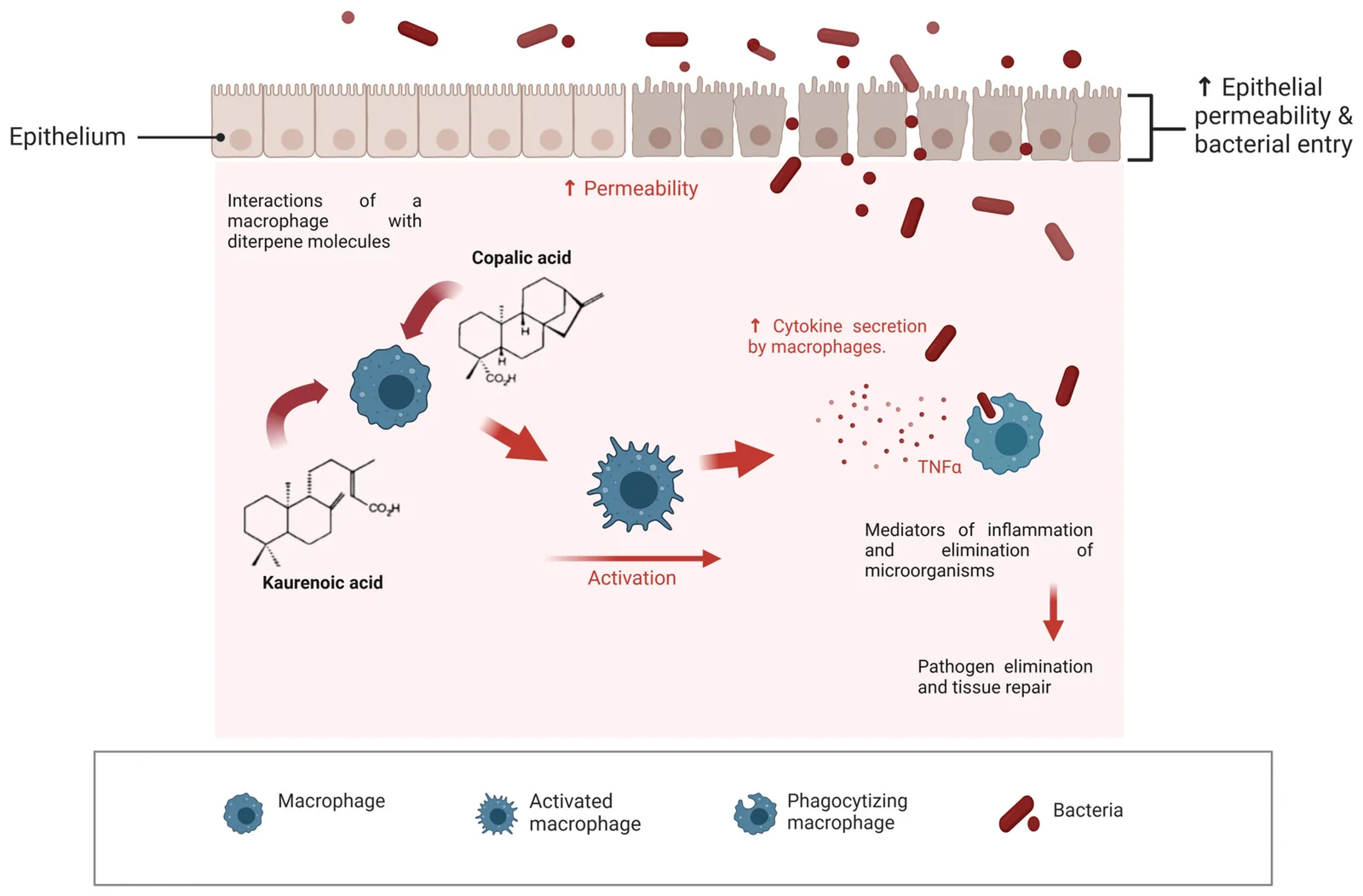
Modulation of Macrophage Activity by Diterpenes. The interaction between diterpenes and macrophages leads to the activation of pro-inflammatory cytokine secretion, with a particular focus on TNF-α. This cytokine plays a fundamental role in initiating the inflammatory response by promoting the recruitment of immune cells and facilitating the phagocytosis of pathogenic microorganisms.

The Figure 3 illustrates how diterpenes modulate the immune response, particularly by activating macrophages and promoting the release of inflammatory cytokines such as TNF-α [44]. These compounds interact with pathogen recognition receptors, like TLRs, on macrophages, triggering a cascade of signals that culminate in the activation of NF-κ, a transcription factor that controls the expression of inflammatory genes. These processes are critical for innate immune responses and the elimination of pathogens and damaged tissues, while also being associated with the control of inflammatory and apoptotic processes [9,73].

Six new diterpenoid compounds were isolated, in addition to nineteen previously known compounds [74]. These compounds demonstrated significant anti-inflammatory activity in the J774A.1 cell line previously stimulated with *Escherichia coli* Lipopolysaccharide (LPS). They inhibited the release of nitric oxide (NO) and the expression of pro-inflammatory enzymes, including cyclooxygenase-2 (COX-2). They showed a significant inhibition of COX-2 formation under inflammatory conditions at all tested concentrations (50–12.5 μM; *p* < 0.001 vs LPS), comparable to indomethacin, a reference drug.

Building on a similar study, Ngo et al. (2022) isolated four previously unknown diterpene compounds, alongside twelve familiar ones, and evaluated their potential to combat inflammation [75]. It was observed that the production of LPS-induced NO in RAW 264.7 cells demonstrated complete suppression with an IC_50_ value of 3.4 ± 1.2 μM, comparable to the positive control, celastrol. To investigate whether these compounds inhibited the expression of inducible nitric oxide synthase (iNOS) and COX-2 at concentrations ranging from 3 to 30 μM, the expression of iNOS and COX-2 was analyzed using the Western blot technique. The results demonstrated that these compounds have the ability to inhibit iNOS protein production; and the intensity of this inhibitory effect is related to the concentration of the compounds used in the experiment.

An in vitro anti-inflammatory analysis of six diterpenic acids demonstrated that only kaurenoic and copalic acids exhibited significant hemolytic activities, reaching 61.7% and 38.4%, respectively, at a concentration of 100 µM [76]. Additionally, only copalic acid (with an inhibition rate of 98.5% ± 1.3%) and hardwickiic acid (with an inhibition rate of 92.7% ± 4.9%) at 100 mM were able to inhibit nitric oxide production in LPS-activated macrophages without affecting the production of TNF-α -. These acids also inhibited the production of IL−6 and increased the production of IL-10. These results clearly demonstrated a therapeutic potential for these diterpenic acids in the treatment of acute injuries, such as inflammation or skin disorders.

A newly discovered diterpene structure, called plebeianiol A, was isolated alongside four previously identified diterpenes [77]. Plebeianiol A displayed notable efficacy in neutralizing the 1,1-diphenyl−2-picrylhydrazyl (DPPH) radical, with IC_50_ values falling within the range of 20.0 to 29.6 µM. Furthermore, it demonstrated significant inhibition of reactive oxygen species (ROS) production in macrophages stimulated by LPS. Additionally, they inhibited NO production in LPS-induced macrophages, with IC_50_ values ranging from 18.0 to 23.6 µM [77]. These findings highlight the therapeutic potential of diterpenes to treat diseases associated with oxidative damage and inflammation. 

Thirteen diterpenoids were identified in the roots of *Euphorbia ebracteolata*, and their structures were determined using techniques such as 1D and 2D Nuclear Magnetic Resonance (NMR), High-Resolution Mass Spectrometry (HRESIMS), and Electronic Circular Dichroism (ECD) [78]. Through the Griess test, it was found that among these compounds, three demonstrated a notable ability to inhibit NO production in LPS-induced macrophages in the RAW 264.7 cell line, with IC_50_ values of 2.44, 2.76, and 1.02 μM, respectively [78]. These results strengthen the potential of diterpenes as anti-inflammatory agents. 

The dipernic hardwickiic acid has been extensively studied for its ability to interact with biological systems, exhibiting antioxidant, antitumor, immunomodulatory, antinociceptive, and anti-inflammatory activities [64]. Its ability to inhibit the production of inflammatory cytokines by suppressing the NF-κB signaling pathway has been demonstrated by luciferase assay [64].

The immunomodulatory effect of kaurenoic acid, a diterpene present in several medicinal plants, was investigated in human monocytes and macrophages [79]. The results showed that kaurenoic acid was able to modulate cytokine production and surface molecule expression in monocytes and macrophages, including a reduction of IL-1β, IL-6, and TNF-α production, and a decrease in CD80 and CD86 expression. Additionally, kaurenoic acid demonstrated antioxidant and anti-inflammatory effects, indicating its therapeutic potential for the treatment of inflammatory and autoimmune diseases.

The diterpenic acid polyaltic acid (AP) was investigated for its potential chemopreventive effects, considering the interplay between inflammatory processes and carcinogenesis [80]. To understand the mechanisms involved in this effect, the anti-inflammatory activity of AP was evaluated in terms of NO and prostaglandin E2 (PGE2) production in rat macrophages. AP reduced LPS-induced NO levels in macrophages, indicating an anti-inflammatory action mediated by the NO pathway. However, this diterpene showed no effect on PGE2 [80]. These results suggest that AP may contribute to the chemopreventive effect in a colon carcinogenesis rat model through its anti-inflammatory activity.

Diterpenoids isolated from H. annuus L. were tested on murine RAW 264.7 macrophages. The study focused on the production of NO, PGE2, and TNF-α. At low concentrations (10 µM), these compounds inhibited NO and PGE2 production in LPS-stimulated macrophages. This inhibition was associated with a concentration-dependent decrease in NOS-2 and COX-2 protein and mRNA expression, suggesting that the diterpenoids suppress NO and PGE2 production by downregulating these enzymes at the transcriptional level [57]. Furthermore, the diterpenoids effectively blocked the release of TNF-α, without causing cell damage. suggesting their potential as safe and effective immunomodulatory agents. 

Overall, diterpenes show promising pharmacological activity as anti-inflammatory agents. However, it is crucial to better characterize their spectrum of immunomodulatory actions and understand the underlying mechanisms to advance their pharmacological potential. Moreover, studies using animal models or clinical trials are scarce, hindering the progress needed to confirm their pharmacological actions and ensure their safety and effectiveness.

### 4.2. Impact of Diterpenes on Humoral and Cellular Immune Responses

Cytokines play a crucial role in inflammatory conditions and as considered key elements in the immune response [81]. These molecules modulate various processes and mechanisms of the cellular and humoral responses within both innate and adaptive pathways. They are crucial for the development and regulation of inflammation pathways [82]. 

**Figure 4:**
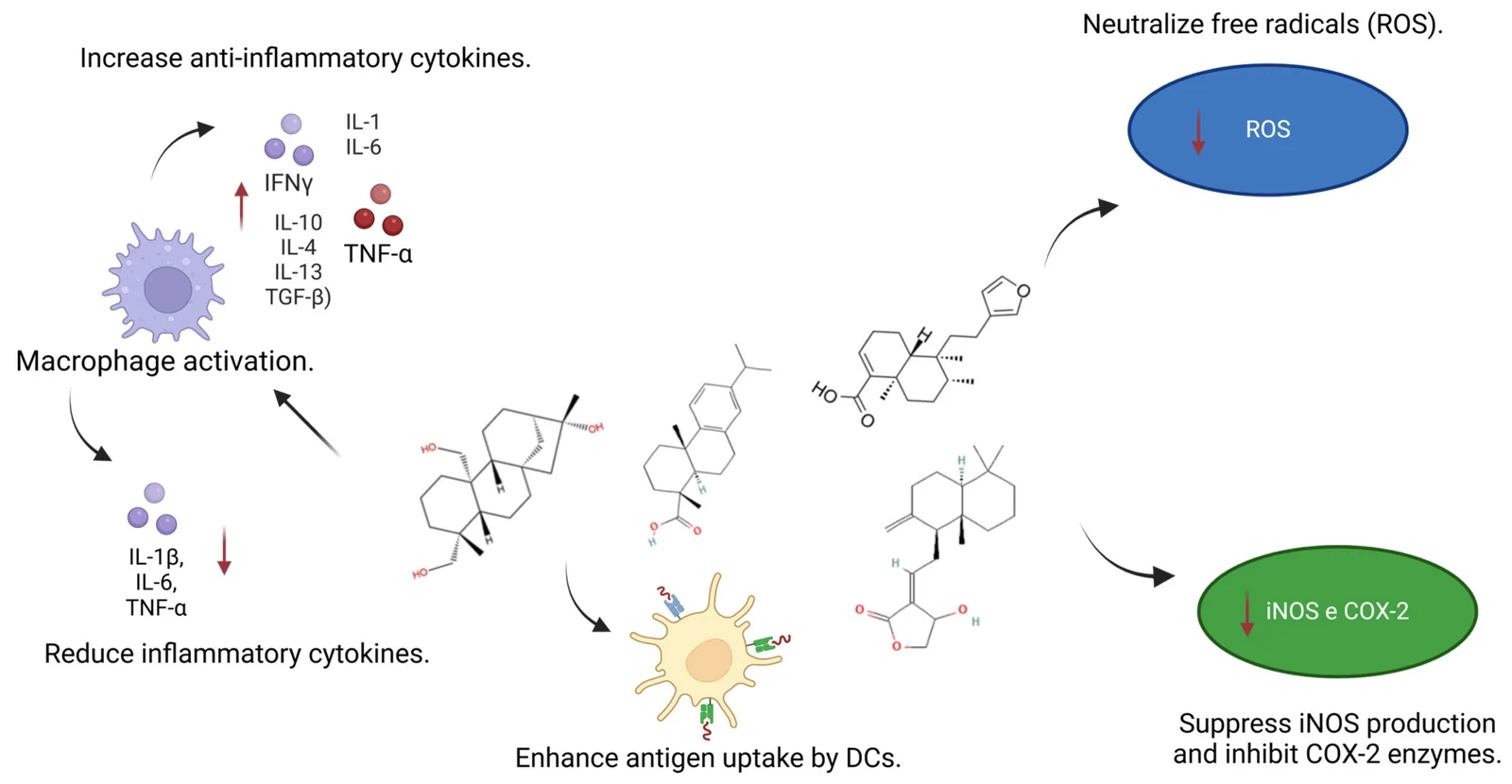
Influence of diterpenes on inflammation dynamic. Diterpenes play a key role in various immune response pathways by regulating the expression and activation of different immune system components. They are particularly involved in balancing pro-inflammatory and anti-inflammatory responses, often promoting an anti-inflammatory state.

Natural products play a promising role in cancer immunotherapy, particularly their ability to modulate the immunosuppressive tumor microenvironment [83]. In this scenario, the dynamic interaction between cytokines and immune cells is essential in regulating the antitumor response, promoting a potential balance between pro-inflammatory and immunosuppressive mechanisms [83]. Pro-inflammatory cytokines, such as IL-1, IL-6, and TNF-α, which signal through type I receptors, exemplify the complexity of the immune signaling network and the necessity for its precise regulation. Figure 4 illustrates how diterpenoid molecules, a type of natural compound, can act as modulators of this signaling, adjusting cytokine expression and promoting targeted recruitment of specific immune cells.

This selective regulation of cellular and molecular interactions prevents excessive inflammatory responses, enhancing antitumor activity and creating an environment less permissive to tumor growth. By fostering a microenvironment more receptive to immunotherapies—such as immune checkpoint inhibitors and cancer vaccines—these natural compounds demonstrate their potential as promising adjuvants in cancer treatment, enabling a more targeted and effective immune response [83].

Maintaining equilibrium in this modulation is vital for sustaining physiological homeostasis and ensuring an appropriate response to infection. The immune system’s response to infection or other stimuli can lead to immunopathologies, primarily due to the overproduction of pro-inflammatory cytokines like TNF-α [84,85,86]. Striking the right balance in this modulation is critical for avoiding potential disruptions in immune function [16,83]. In this context, the overproduction of pro-inflammatory cytokines can result in chronic inflammation and healthy tissues damage [16,83].

Conversely, insufficient cytokine production can lead to a compromised immune response, rendering the body vulnerable to unchecked infections [83,87]. If there is insufficient production of these cytokines, the immune system may not be able to mount an effective response against invading pathogens, rendering the body vulnerable to severe infections [83,87].

The immunomodulation induced by natural compounds involves altering the equilibrium among various subsets of immune cells. This sophisticated process entails a complex interplay among opposite forces. On one side, pathogenic cells drive inflammation, potentially harming the body. On the other side, protective cells act as firefighters, quelling inflammation and regulating the immune response. By influencing the cytokines expression in different cell populations, natural compounds can tip the scales, promoting health and resilience [87,88].

Different diterpenoids isolated from *Hemionitis albofusca*, specifically the compounds (A) 14-oxy-7β,20-dihydroxycylate-12,18-diene; (B) ent-8(14),15-pimaradiene-2β,19-diol; (C) ent-kaurane-16-ene-2β,18α-diol; and (D) ent-kaurane−2β,16α,18α-triol, have demonstrated various biological activities that influence multiple aspects of the immune response [65]. These natural molecules were able to modulate nitric oxide (NO) production and the expression of inflammatory markers such as iNOS, TNF-α, and IL-6 in lipopolysaccharide-induced RAW264.7 cells. Additionally, the diterpenoids showed the ability to inhibit p38 protein phosphorylation, interfering with the p38 MAPK signaling pathway. The activation of the p38 MAPK signaling pathway is associated with a variety of biological processes, including inflammation, cell proliferation, differentiation, and apoptosis. This natural compound-mediated immunomodulation process can encompass a range of effects, including the suppression of exacerbated inflammatory responses and the promotion of regulatory immune responses [87,88].

Simultaneously, it is observed that these natural compounds may play a significant role in promoting the function of regulatory cells in the immune system [89]. A study observed that diterpenoid compounds, such as neophytadiene, demonstrated cytotoxic activity against A549 (lung cancer) and PC−3 (prostatic adenocarcinoma) cancer cells, suggesting a potential role in inducing apoptosis in these cells [90].

Additionally, it has been discussed that these compounds may have a significant role in promoting the function of regulatory cells in the immune system, such as regulatory T (Tregs) and Th17 cells [89]. Tregs play an essential role in modulating the immune environment, controlling autoimmune responses, and ensuring immune tolerance. The ability of these natural compounds to favor Tregs activity is of great relevance, as it contributes to maintaining immune balance, preventing uncontrolled immune reactions, and autoimmunity [91]. Th17 cells are CD4^+^ T lymphocytes that are essential for defense against extracellular pathogens, promoting inflammation through the secretion of IL−17 [92]. However, their dysregulation is associated with autoimmune diseases, such as rheumatoid arthritis and multiple sclerosis. 

The balance between Tregs and Th17 is essential for the regulation of the immune response, and diterpenes capable of modulating this interaction have relevant therapeutic potential. In this context, the diterpene triptolide, a natural compound isolated from the plant Trip-terygium wilfordii, has demonstrated immunosuppressive effects in inflammatory and autoimmune diseases. Studies indicate that it can suppress collagen-induced rheumatoid arthritis by inhibiting Th17 cell differentiation and regulating CD4^+^ and CD8^+^ lymphocytes in Peyer’s patches [93,94,95]. Other studies also show that triptolide is capable of acting in the improvement of systemic lupus erythematosus by upregulating Tregs and the expression of miR-125a-5p, in addition to inhibiting the formation of Th17 by inhibiting the AKT/mTOR/p70S6K pathway in vitro and in vivo [96,97].

This complex process of cellular modulation highlights the potential therapeutic applicability of these compounds in the fine and adaptive regulation of the immune system [89]. Natural products and their compounds may modulate the production of immune mediators through different ways. *Cymbopogon citratus* and its isolated compound citral were found to stimulate the production of IL-1β and IL-6 without affecting the production of IL-10 in murine macrophages in vitro. This effect was observed in the context of cytokine production by macrophages challenged with lipopolysaccharide (LPS), without affecting the production of IL-10 [98]. 

*Copaifera* (*Copaifera* spp.) oleoresins have been shown to induce human monocyte activation without compromising cell viability. The results suggest that diterpenic or sesquiterpenic acids present in the oleoresins may be involved in the mechanisms underlying this activation [16]. However, it is not clear how this compound affects the production of other inflammatory mediators in monocytes. To fully understand how oleoresins work, we need to delve deeper into two key areas: their impact on immune cells’ ability to kill microbes and how they affect cell receptors. This would provide valuable insights into the mechanisms behind their action. Specifically, further studies are crucial to pinpoint the exact effects of diterpenes and sesquiterpenes, the active compounds in Copaiba oleoresin, on monocytes and other immune cells. 

Abietan diterpenes isolated from *Plectranthus grandidentatus* exhibit an inhibitory effect on mitogen-induced T and B lymphocyte proliferation, with an emphasis on T cells [98]. This effect is mediated by the ability of these diterpenes to reduce CD69 expression and induce lymphocyte apoptosis. Furthermore, an immunosuppressive activity of diterpenoid compounds from *Cephalotaxus fortunei var. alpina* and *C. sinensis* has been reported, inhibiting the proliferation of T and B cells [99]. Similarly, the authors of [100] isolated diterpene cascarinoids from *Croton cascarilloides*, which showed immunosuppressive activity against the proliferation of both B and T cells in vitro. These compounds inhibited LPS-induced B cell proliferation and ConA-induced T cell proliferation. 

The immunosuppression of these lymphocytes occurs due to the ability of cascarinoids to modulate the immune response, interfering with cell proliferation processes and B and T cell activation [100]. This immunosuppressive activity may be associated with the inhibition of the expression of pro-inflammatory cytokines, such as IL-2, involved in the activation and proliferation of T lymphocytes [101]. Diterpenes may also act by modulating levels of cyclic adenosine 3′,5′-monophosphate (cAMP), a molecule involved in regulating lymphocyte growth and differentiation. 

For example, forskolin (Fsk) modulates B cell activation by regulating plasma cAMP levels and selectively inhibiting the progression of the G1 to S phase transition induced by B cell growth factor (BCGF) [102,103]. It also regulates T cell activation by elevating intracellular cAMP levels, promoting their differentiation into Th1 and Th2 phenotypes, while significantly suppressing the Th17 profile [104]. Furthermore, Fsk inhibits human T cell proliferation by downregulating IL-2 signaling by blocking Stat5a/b phosphorylation, IL-2R complex formation, and PKA-mediated Jak3 activation [105]. These findings highlight the interplay between the Jak3/Stat5 and cAMP/PKA pathways in modulating T cell function and proliferation mediated by the IL-2 receptor (IL-2R ). Finally, modulation of intracellular cAMP levels occurs through activation of adenylyl cyclases (ACs 1–8) by Fsk [106].

Regulation of the adaptive immune response and interference in the cellular signaling necessary for the activation and proliferation of lymphocytes are also factors that may be associated [107]. Understanding these mechanisms of immunosuppression is crucial for exploring the therapeutic potential of cascarinoids A and B and developing strategies to selectively modulate the immune response in pathological conditions, where exacerbated immune response may be detrimental [100]. 

Such disparities in the results point to the diversity of cellular responses triggered by different classes of diterpenoids, consequently emphasizing the need for further investigations for a deeper understanding of the therapeutic properties of these compounds. The study [108] on daphnane diterpenes from the latex of *Hura crepitans* L. also highlights the diversity of cellular responses induced by different classes of diterpenoids, specifically regarding the antiproliferative activity in colorectal cancer cells. The results indicate that huratoxin and 4′,5′-epoxyhuratoxin show significant and selective inhibition of cell growth against the colorectal cancer cell line Caco-2 and primary colorectal cancer cells cultured as colonoids.

The ethanol (EtOH) extracts and andrographolides, purified diterpenes from the plant *Andrographis paniculata*, have demonstrated the ability to stimulate the immune response in in vitro and in vivo models, evidenced by a remarkable induction of IL-2 production, antibodies, and a delayed hypersensitivity response to antigens [109,110]. In addition to these effects, the plant preparations also triggered a nonspecific immune response in mice, involving macrophage migration, efficient phagocytosis of bacteria, and proliferation of splenic lymphocytes [110]. These results point to a comprehensive modulation of the immune response, addressing both specific and nonspecific components of the immune system [110].

It is important to note that, although both andrographolide and EtOH extract contributed to the stimulation of the immune response, it was observed that the specific immune response was less intense with andrographolide compared to the EtOH extract [110]. This observation suggests that other substances present in the extract may play an additional role in immunostimulation, highlighting the complexity and synergy of compounds in preparations of *Andrographis paniculata*.

The methanolic extract derived from the rhizome of *Hedychium coronarium* exhibits remarkable inhibitory properties on the increased vascular permeability induced by acetic acid in mice, as well as on the production of nitric oxide in lipopolysaccharide-activated mouse peritoneal macrophages [111]. From this extract, three new labdane-type diterpenes, named hediquilactones A, B, and C, were isolated, along with six known diterpenes. The structural elucidation of hediquilactones was based on chemical and physicochemical evidence [98]. The diterpene constituents showed notable inhibitory effects in assays related to increased vascular permeability. Additionally, they demonstrated the ability to inhibit nitric oxide production and the induction of nitric oxide synthase in activated peritoneal macrophages. These findings highlight the therapeutic potential of diterpenes in modulating inflammatory responses and regulating vascular events, suggesting potential pharmacological applications derived from the plant *Hedychium coronarium* [111].

Therefore, diterpenoids, such as those isolated from Hemionitis albofusca, have therapeutic potential for inflammatory and autoimmune diseases, such as rheumatoid arthritis, multiple sclerosis, lupus, and inflammatory bowel disease. They modulate the immune response by inhibiting Th17 cell differentiation, promoting Treg activity, suppressing pro-inflammatory cytokines (TNF-α, IL-6) and blocking signaling pathways such as p38 MAPK. These mechanisms help to control chronic inflammation and exacerbated autoimmune responses, highlighting their role in restoring immune balance.

### 4.3. The Influence of Diterpenes on Inflammasome Pathways

Inflammasomes are multiprotein complexes that play a central role in regulating the innate immune response, acting as sensors for danger signals and pathogens. However, their dysregulation is closely associated with various inflammatory conditions [112,113]. Inappropriate activation of inflammasomes can trigger excessive release of pro-inflammatory interleukins, such as IL-1β, and induce pyroptosis, a specific form of inflammatory cell death. These exacerbated processes significantly contribute to the pathogenesis of multiple inflammatory diseases [112]. Conditions associated with uncontrolled inflammasome activation include cardiovascular disorders, neurodegenerative diseases, autoinflammatory syndromes, kidney disorders, gastrointestinal diseases, joint diseases, and dermatological conditions, among others [112]. Thus, precise regulation of inflammasomes is essential to prevent and effectively treat a diverse range of inflammatory diseases, aiming for selective modulation of these inflammatory pathways without compromising innate immunity.

Nucleotide-binding domain and leucine-rich protein (NLRP) family inflammasomes, especially NLRP3, play a significant role in several pathologies [9]. Several diterpenes, such as andrographolide, rosthornin B, triptolide, caurenoic acid, carnosic acid, oridonin, teuvincenone F, and derivatives of tanshinone IIA and phorbol, were indicated as potential candidates for treating NLRP3-mediated inflammatory diseases due to their effectiveness in modulating the NLRP3 pathway [9,114].

**Figure 5:**
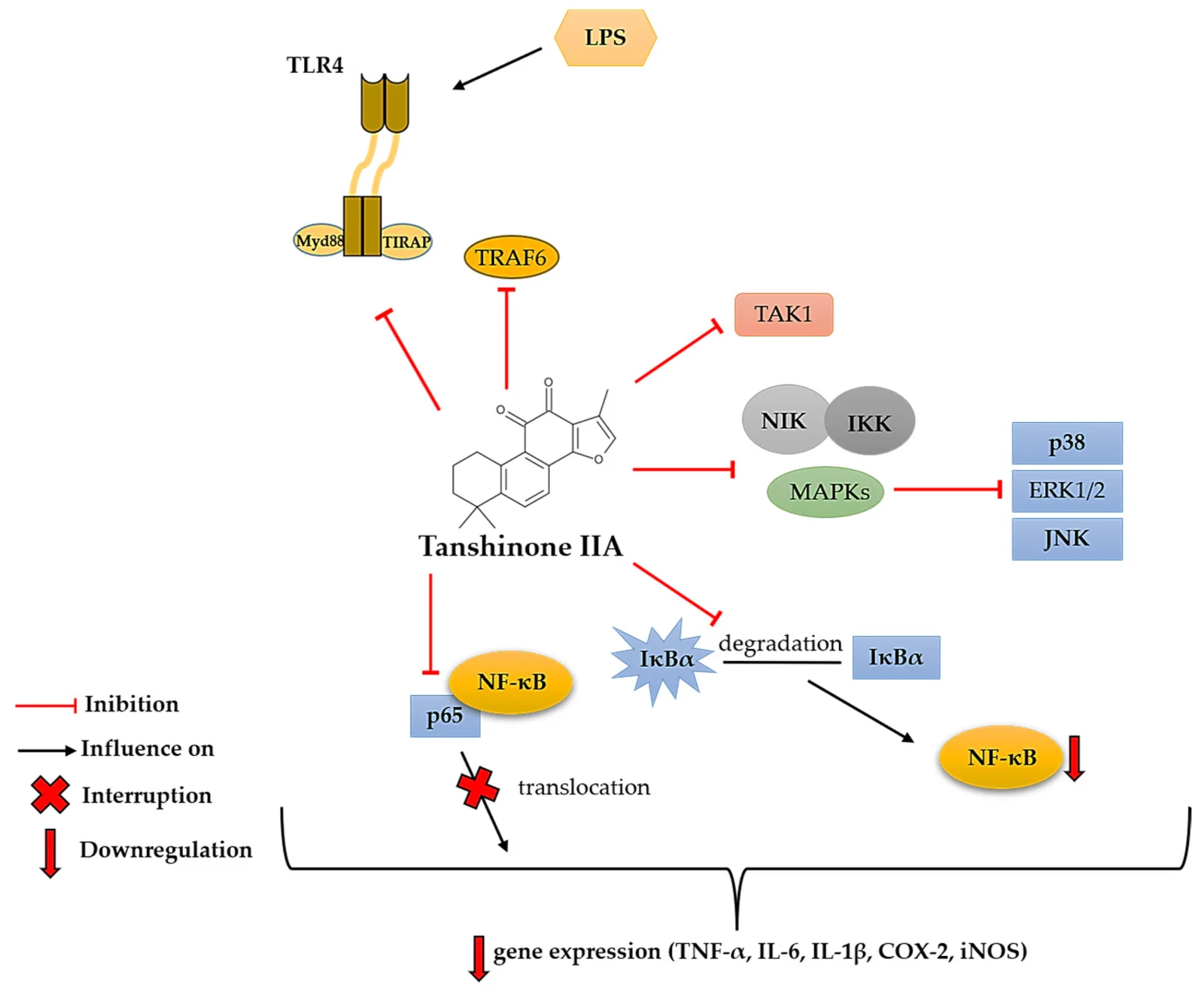
Mechanism of anti-inflammatory action of tanshinone IIA (Tan IIA) on the NF-κB pathway. Tan IIA suppresses NF-κB signaling in LPS-stimulated cells by inhibiting IκBα degradation, the NIK-IKK pathway, and the MAPKs (p38, ERK1/2, JNK) pathways. Furthermore, it inhibits TLR4 expression, suppresses adaptor molecules (MyD88, TRAF6), blocks TAK1 phosphorylation, and prevents p65 nuclear translocation. These effects reduce the expression of pro-inflammatory genes (TNF-α, IL-6, IL-1β, COX-2, iNOS), highlighting its therapeutic potential in controlling inflammation.

Tanshinone IIA (Tan IIA), a bioactive diterpene derived from the roots of Salvia miltiorrhiza Bunge (Labiatae), has recognized anti-inflammatory properties, acting predominantly on the NF-κB pathway, a key regulator of inflammasome activation (Figure 5) [75]. In LPS-stimulated Raw 264.7 cells, it suppresses the NF-κB signaling pathway by inhibiting IkappaB alpha degradation and suppressing the NIK-IKK pathway as well as the MAPKs (p38, ERK1/2, and JNK) pathway. Furthermore, Tan IIA inhibits the expression of TLR4, suppresses upstream adaptor molecules such as MyD88 and TRAF6, inhibits the phosphorylation of TAK1 and the nuclear translocation of the p65 subunit [115,116,117]. These mechanisms result in reduced expression of pro-inflammatory genes such as TNF-α, IL-6, IL-1β, COX-2 and iNOS, highlighting the therapeutic potential of Tan IIA in the treatment of inflammatory and autoimmune diseases, including rheumatoid arthritis, atherosclerosis, neuroinflammation, cardiovascular diseases, and gastric cancer [118,119].

A study investigated the effect of Tan IIA and the underlying mechanism on the progression of diabetic nephropathy in vitro [120]. The results indicated that Tan IIA downregulates the expression of transforming growth factor beta 1 (TGF-β1), inhibiting the pyroptosis of renal tubular epithelial cells (HK−2) induced by high glucose concentration. In vivo, it reduces the inflammatory response of macrophages induced by LPS by suppressing the induction of HIF−1α via inactivation of succinate dehydrogenase (SDH), in addition to preserving Sirt2 activity through downregulation of glycolysis, contributing to the inhibition of the NLRP3 inflammasome [121]. Additionally, Tan IIA demonstrated cardioprotective effects by inhibiting the activation of the NLRP3 inflammasome and modulating the differentiation of Th17/Treg cells, highlighting itself as a potential therapeutic agent for inflammatory diseases associated with the heart [122].

The diterpene compounds phytanol and phytanyl amine have been shown to promote the reduction of NLRP3 expression, as well as inflammatory mediators and chemotactic proteins in BALC/c mice [123]. Additionally, a decrease in the release of B lymphocyte chemotactic chemokine (BLC), T-cell activating chemokines-3, tricarboxylic acid (TCA), IL-4, IL-12, and the tissue inhibitor of metalloproteinase−1 (TIMP-1) was observed. These results highlight the potential of phytanol and phytanyl amine as modulatory agents for inflammatory and immunological conditions, particularly in reducing NLRP3-mediated inflammatory responses. This suggests promising therapeutic applications for the treatment of inflammatory and immunological disorders.

The diterpene triptolide has the ability to reduce serum levels of pro-inflammatory cytokines, including IL-1β and IL-18, as well as the expression of NLRP3 and toll-like receptor 4 (TLR4) in animal models [102]. This compound has the potential to prevent the progression of inflammatory diseases such as immunoglobulin A (IgA) nephropathy and aortic transverse constriction-induced myocardial remodeling [124]. Additionally, this compound can inhibit NLRP3 and the pro-fibrotic pathway of transforming growth factor beta 1 (TGF-β1) and negatively regulate NLRP3 by targeting hsa-miR-20b (microRNA) [37,125]. In a gastric cancer model, triptolide demonstrated the ability to inhibit IL−8 expression induced by IL-1β, in addition to suppressing the activation of the AP-1 axes, mediated by ROS/ERK, and NF-κB, mediated by ROS [126].

**Figure 6:**
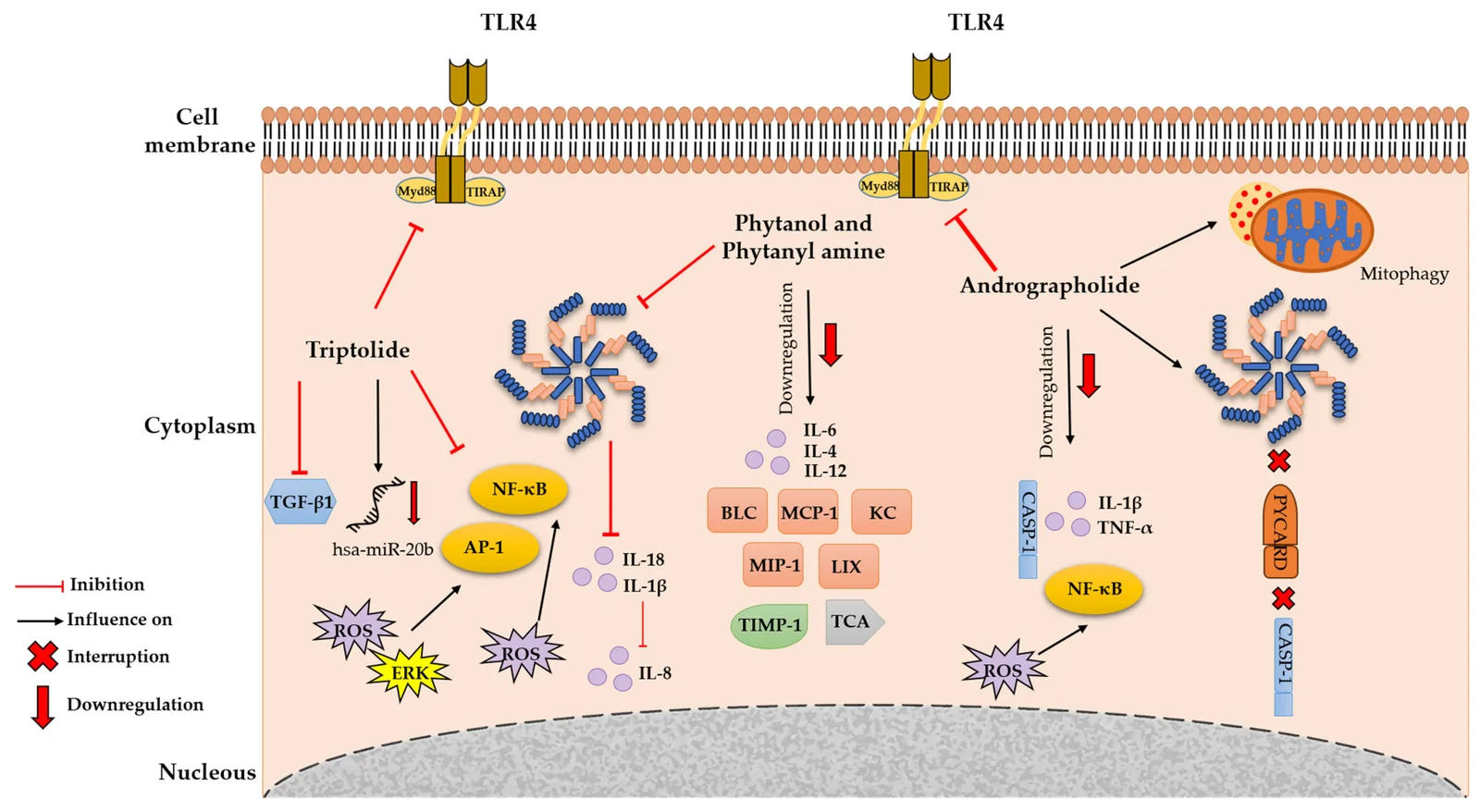
Mechanism of anti-inflammatory action of diterpenes focusing on the NLRP3 inflammasome. Triptolide inhibits the TLR4 pathway and NLRP3 inflammasome activation, reducing the release of pro-inflammatory cytokines (IL-1β and IL-18), suppressing TGF-β1 expression, regulating microRNAs (such as hsa-miR-20b), and inhibiting IL-1β-induced IL-8 expression. Furthermore, it blocks the activation of the AP-1 (ROS/ERK-mediated) and NF-κB (ROS-mediated) axes. Phytanol and phytanylamine suppress the expression of NLRP3, inflammatory interleukins (IL-6, IL-4, IL-12), chemokines (MCP-1, MIP-1, BLC) and TIMP-1, demonstrating anti-inflammatory and immunomodulatory properties. Andrographolide prevents the formation of the NLRP3-PYCARD-CASP1 complex, reduces inflammatory mediators such as TNF-α, IL-1β and ROS/NF-κB, and stimulates mitophagy, contributing to cellular protection and inflammation reduction.

The anti-inflammatory property of triptolide manifests through the inhibition of excessive NLRP3 inflammasome activation (Figure 6). This specific molecular action culminates in the subsequent reduction of the inflammatory response associated with specific pathological conditions [104]. In particular, Triptolide’s ability to modulate NLRP3 suggests potential applicability in the treatment of inflammatory diseases where chronic inflammation plays a prominent role. Additionally, Triptolide’s role in attenuating cardiac fibrosis is highlighted [22,127]. Cardiac fibrosis, a process characterized by the replacement of normal cardiac tissue with scar tissue, often results from a chronic inflammatory response. By modulating NLRP3, Triptolide presents therapeutic potential by interrupting or slowing the course of cardiac fibrosis, mitigating the underlying inflammatory response [127].

Andrographolide is a bitter diterpene lactone with anticolitis and antitumor effects. This compound reduces the expression of cleaved CASP1, IL-1β, and the collapse of the mitochondrial membrane potential through the PIK3CA-AKT1-MTOR-RPS6KB1 pathway (Figure 6) [128]. It also inactivates the NLRP3 inflammasome, induces autophagy, and enhances the disruption of the NLRP3-PYCARD-CASP1 complex and mitophagy in mouse macrophages [128]. Furthermore, it negatively regulates the expression of TNF-α, IL-1β, and NLRP3 and suppresses ROS-mediated NF-κB expression in female C57/BL6 mice induced by ovalbumin (OVA) and bone marrow-derived macrophages (BMDM) (primary culture of murine bone marrow-derived macrophages) [34]. Another study also demonstrated that this diterpene has inhibitory effects on gene expression and protein levels of NLRP3, Caspase-1 and IL-1β, with molecular docking analyses indicating high binding affinity to targets of this pathway (Figure 6) [129]. 

Andrographolide also exhibits a hepatoprotective effect in mice with choline deficiency, reducing hepatic inflammation and fibrosis [130]. Also, andrographolide reduces the overexpression of HMGB1, TLR4, NF-κB, COX−2, iNOS, and NLRP3, inhibits the overexpression of MIP−1α and P2X7 receptor, and modulates the expression of protein markers in mouse glial cells (Figure 6) [130]. 

Thus, andrographolide and other diterpenes discussed in this section demonstrate a significant capacity to inhibit the NF-κB pathway and, consequently, the activation of inflammasomes. This property gives these compounds a high therapeutic potential for the treatment of several inflammatory diseases, including asthma, lung lesions and chronic colitis, as well as autoimmune diseases, such as systemic lupus erythematosus (SLE), and malignant neoplasms, such as metastatic luminal breast cancer [131,132,133,134,135]

Although these results provide a comprehensive insight into the role of diterpenes in modulating the NLRP3 inflammasome and their potential therapeutic applications in in-flammatory and immunological conditions, it is concluded that they have the potential to modulate the NLRP3 inflammasome and attenuate the inflammatory response in various inflammatory and autoimmune diseases. However, further research is needed to fully understand their mechanisms of action and efficacy in specific conditions. 

Addressing gaps in the understanding of these mechanisms and conducting additional clinical studies to assess the safety and efficacy of diterpenes in patients with inflammatory diseases are crucial. Furthermore, clarity in discussing the mechanisms of action and potential side effects is essential to improve the understanding and clinical applicability of these compounds.

## 5. Conclusions

Diterpenes are promising therapeutic alternatives for interventions targeting immune dysfunctions and inflammatory diseases due to their diverse immunomodulatory activities. These compounds regulate cytokine expression, modulate immune cell response, and suppress inflammatory mediators. These activities hold potential therapeutic applications for conditions such as tuberculosis, autoimmune diseases, and cancer. Their ability to influence inflammasome pathways, especially NLRP3, has shown effectiveness in reducing excessive inflammation and mitigating chronic inflammatory conditions such as IgA nephropathy, cardiac fibrosis, and colitis. Additionally, diterpenes exhibit versatile pharmacological properties, including anti-inflammatory, antimicrobial, and anticancer effects, underscoring their therapeutic value in various clinical contexts.

Although diterpenes have significant potential in modulating the immune system, substantial gaps remain in our understanding of their immunomodulatory mechanisms. Further research is essential to investigate their interactions with various immune cells, the cellular signaling pathways involved, and their overall mechanisms of action. Additionally, exploring the synergistic effects of diterpenes in plant extracts could enhance their immunomodulatory activities.

To advance the investigation of diterpenes’ therapeutic potential, it’s essential to conduct studies using in vivo models, including human trials. The limited research in this area has led to significant uncertainties regarding the optimal dosages, formulations, and administration routes for clinical use. Well-designed clinical trials are necessary to evaluate the efficacy of diterpenes in treating various immune-related conditions, such as autoimmune diseases, allergies, and chronic inflammatory disorders. These studies will provide crucial insights into whether the immunoregulatory benefits observed in animal models can be reliably replicated in humans. Therefore, a thorough exploration of diterpenes’ mechanisms and effects is vital to unlocking their full immunomodulatory potential for therapeutic applications.

## Figures and Tables

**Table 1:** Anti-Inflammatory activity of diterpenes.

Species	Compounds	Structure	Action	Reference
*Isodon henryi*; *Gymnocoronis spilanthoides* var.	ent-kaurane	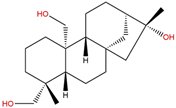	Inhibition of nitric oxide production; increased production of IL−10	[62,63]
*Copaifera pubiflora*	ent-hardwickiic acid	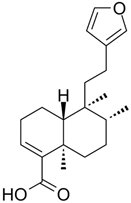	Suppression of the NF-κB signaling pathway	[64]
*Hemionitis albofusca*	Onychiol B	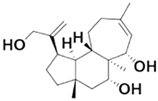	Inhibition of nitric oxide production; inhibition of TNF-α and IL−6	[65,66]
*Pinus pinaster*	Dehydroabietic acid	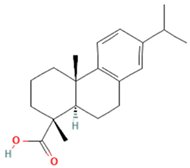	NF-κB inhibition; Inhibition of nitric oxide production	[67,68]
*Amomum villosum*	isocoronarin D	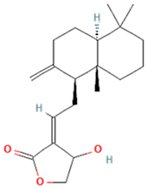	Inhibition of COX2 and NOS2; NF-κB inhibition	[69]
*Sigesbeckia glabrescens*	Darutigenol	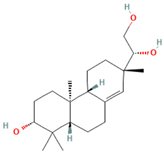	NF-κB inhibition	[70]
*Euphorbia wallichii*	Jolkinolide B	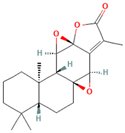	Inhibition of nitric oxide production	[71]
*Euphorbia myrsinites*	Myrsatisane	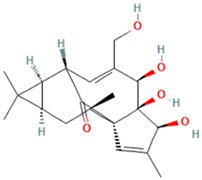	Inhibition of nitric oxide production; inhibition of IL−1β	[72]

## References

[1] Liu Y., Chen X., Zhang C. (2023). Sustainable Biosynthesis of Valuable Diterpenes in Microbes. Eng. Microbiol..

[2] Liu Y.-F., Yang B.-C., Song Z.-M., Qiao L.-Q., Peng R., Feng W.-S., Cheng Y.-X., Wang Y.-Z. (2023). Seven Diterpenoids from the Resin of *Pinus Yunnanensis* Franch and Their Anti-Inflammatory Activity. Fitoterapia.

[3] Antoine G., Vaissayre V., Meile J.-C., Payet J., Conéjéro G., Costet L., Fock-Bastide I., Joët T., Dussert S. (2023). Diterpenes of *Coffea* Seeds Show Antifungal and Anti-Insect Activities and Are Transferred from the Endosperm to the Seedling after Germination. Plant Physiol. Biochem..

[4] Ren J., Wu Y., Zhu Z., Chen R., Zhang L. (2022). Biosynthesis and Regulation of Diterpenoids in Medicinal Plants. Chin. J. Nat. Med..

[5] Gómez-Hurtado M.A., Nava-Andrade K., Villagómez-Guzmán A.K., del Río R.E., Andrade-López N., Alvarado-Rodríguez J.G., Martínez-Otero D., Morales-Morales D., Rodríguez-García G. (2017). Facile Synthesis and Structural Characterization of *μ*4-Oxo Tetrazinc Clusters of Beyerenoic and Kaurenoic Acids. Tetrahedron Lett..

[6] Eksi G., Kurbanoglu S., Erdem S.A., Sanches Silva A., Nabavi S.F., Saeedi M., Nabavi S.M. (2020). Chapter 9—Analysis of Diterpenes and Diterpenoids. Recent Advances in Natural Products Analysis.

[7] Gazim Z.C., Rodrigues F., Amorin A.C.L., Rezende C.M.d., Soković M., Tešević V., Vučković I., Krstić G., Cortez L.E.R., Colauto N.B. (2014). New Natural Diterpene-Type Abietane from *Tetradenia riparia* Essential Oil with Cytotoxic and Antioxidant Activities. Molecules.

[8] Jing W., Zhang X., Zhou H., Wang Y., Yang M., Long L., Gao H. (2019). Naturally Occurring Cassane Diterpenoids (CAs) of *Caesalpinia*: A Systematic Review of Its Biosynthesis, Chemistry and Pharmacology. Fitoterapia.

[9] Islam M.T., Bardaweel S.K., Mubarak M.S., Koch W., Gaweł-Beben K., Antosiewicz B., Sharifi-Rad J. (2020). Immunomodulatory Effects of Diterpenes and Their Derivatives Through NLRP3 Inflammasome Pathway: A Review. Front. Immunol..

[10] Yue G.G.-L., Liang X.-X., Li X.-L., Lee J.K.-M., Gao S., Kwok H.-F., Lau C.B.-S., Xiao W.-L. (2020). Immunomodulatory and Antitumour Bioactive Labdane Diterpenoids from *Leonurus japonicus*. J. Pharm. Pharmacol..

[11] Jantan I., Ahmad W., Bukhari S.N.A. (2015). Plant-Derived Immunomodulators: An Insight on Their Preclinical Evaluation and Clinical Trials. Front. Plant Sci..

[12] Mechchate H., Es-safi I., Jawhari F.z., Bari A., Grafov A., Bousta D. (2020). Ethnobotanical Survey about the Management of Diabetes with Medicinal Plants Used by Diabetic Patient in Region of Fez-Meknes, Morocco. Ethnobot. Res. Appl..

[13] Ali Reza A.S.M., Nasrin M.S., Hossen M.A., Rahman M.A., Jantan I., Haque M.A., Sobarzo-Sánchez E. (2023). Mechanistic Insight into Immunomodulatory Effects of Food-Functioned Plant Secondary Metabolites. Crit. Rev. Food Sci. Nutr..

[14] Kim T., Song B., Cho K.S., Lee I.-S. (2020). Therapeutic Potential of Volatile Terpenes and Terpenoids from Forests for Inflammatory Diseases. Int. J. Mol. Sci..

[15] Cao Y., Feng Y.-H., Gao L.-W., Li X.-Y., Jin Q.-X., Wang Y.-Y., Xu Y.-Y., Jin F., Lu S.-L., Wei M.-J. (2019). Artemisinin Enhances the Anti-Tumor Immune Response in 4T1 Breast Cancer Cells in Vitro and in Vivo. Int. Immunopharmacol..

[16] Santiago M.B., dos Santos V.C.O., Teixeira S.C., Silva N.B.S., de Oliveira P.F., Ozelin S.D., Furtado R.A., Tavares D.C., Ambrósio S.R., Veneziani R.C.S. (2023). Polyalthic Acid from *Copaifera lucens* Demonstrates Anticariogenic and Antiparasitic Properties for Safe Use. Pharmaceuticals.

[17] Pinzi L., Rastelli G. (2019). Molecular Docking: Shifting Paradigms in Drug Discovery. Int. J. Mol. Sci..

[18] Fedorova V.A., Kadyrova R.A., Slita A.V., Muryleva A.A., Petrova P.R., Kovalskaya A.V., Lobov A.N., Zileeva Z.R., Tsypyshev D.O., Borisevich S.S. (2021). Antiviral Activity of Amides and Carboxamides of Quinolizidine Alkaloid (-)-Cytisine against Human Influenza Virus A (H1N1) and Parainfluenza Virus Type 3. Nat. Prod. Res..

[19] Ferreira L.G., Santos R.N.d., Oliva G., Andricopulo A.D. (2015). Molecular Docking and Structure-Based Drug Design Strategies. Molecules.

[20] de Sousa L.R.F., Wu H., Nebo L., Fernandes J.B., da Silva M.F.d.G.F., Kiefer W., Kanitz M., Bodem J., Diederich W.E., Schirmeister T. (2015). Flavonoids as Noncompetitive Inhibitors of Dengue Virus NS2B-NS3 Protease: Inhibition Kinetics and Docking Studies. Bioorg. Med. Chem..

[21] Cabarcas-Montalvo M., Maldonado-Rojas W., Montes-Grajales D., Bertel-Sevilla A., Wagner-Döbler I., Sztajer H., Reck M., Flechas-Alarcon M., Ocazionez R., Olivero-Verbel J. (2016). Discovery of Antiviral Molecules for Dengue: In Silico Search and Biological Evaluation. Eur. J. Med. Chem..

[22] Li Z., Wang J., Cheng X., Hu H., Guo C., Huang J., Chen Z., Lu J. (2021). The Worldwide Seroprevalence of DENV, CHIKV and ZIKV Infection: A Systematic Review and Meta-Analysis. PLoS Negl. Trop. Dis..

[23] Xiao Y., Zhang Y., Ji W.-S., Jia X.-N., Shan L.-H., Li X., Liu Y.-J., Jiang T., Gao F. (2023). Discovery of Myrsinane-Type *Euphorbia* Diterpene Derivatives through a Skeleton Conversion Strategy from Lathyrane Diterpene for the Treatment of Alzheimer’s Disease. Bioorganic Chem..

[24] Wong K.K.-K., Ngo J.C.-K., Liu S., Lin H., Hu C., Shaw P.-C., Wan D.C.-C. (2010). Interaction Study of Two Diterpenes, Cryptotanshinone and Dihydrotanshinone, to Human Acetylcholinesterase and Butyrylcholinesterase by Molecular Docking and Kinetic Analysis. Chem. Biol. Interact..

[25] Doorandishan M., Pirhadi S., Swilam M.M., Gholami M., Ebrahimi P., El-Seedi H.R., Jassbi A.R. (2021). Molecular Docking and Simulation Studies of a Novel Labdane Type- Diterpene from *Moluccella aucheri* Scheen (*Syn. Otostegia aucheri*) as Human- AChE Inhibitor. J. Mol. Struct..

[26] Hu G., Peng X., Dong D., Nian Y., Gao Y., Wang X., Hong D., Qiu M. (2021). New *Ent*-Kaurane Diterpenes from the Roasted Arabica Coffee Beans and Molecular Docking to *α*-Glucosidase. Food Chem..

[27] Phong N.V., Trang N.M., Quyen C.T., Anh H.L.T., Vinh L.B. (2022). SARS-CoV-2 Main Protease and Papain-like Protease Inhibition by Abietane-Type Diterpenes Isolated from the Branches of *Glyptostrobus pensilis* Using Molecular Docking Studies. Nat. Prod. Res..

[28] Feng H., Jiang Y., Cao H., Shu Y., Yang X., Zhu D., Shao M. (2022). Chemical Characteristics of the Sesquiterpenes and Diterpenes from Lauraceae Family and Their Multifaceted Health Benefits: A Review. Heliyon.

[29] Zhang P., Xue S., Tang P., Cui Z., Wang Z., Luo J., Kong L. (2021). Aphamines A–C, Dimeric Acyclic Diterpene Enantiomers from *Aphanamixis polystachya*. Chin. Chem. Lett..

[30] Thakor P., Subramanian R.B., Thakkar S.S., Ray A., Thakkar V.R. (2017). Phytol Induces ROS Mediated Apoptosis by Induction of Caspase 9 and 3 through Activation of TRAIL, FAS and TNF Receptors and Inhibits Tumor Progression Factor Glucose 6 Phosphate Dehydrogenase in Lung Carcinoma Cell Line (A549). Biomed. Pharmacother. Biomed. Pharmacother..

[31] Fang F.-H., Huang W.-J., Zhou S.-Y., Han Z.-Z., Li M.-Y., Liu L.-F., Wu X.-Z., Yao X.-J., Li Y., Yuan C.-S. (2017). Aphapolins A and B: Two Nemoralisin Diterpenoids Isolated from *Aphanamixis polystachya* (Wall.) R. Parker. Eur. J. Org. Chem..

[32] Gao X., He J., Wu X.-D., Peng L.-Y., Shao L.-D., Li Y., Cheng X., Zhao Q.-S. (2017). Sauruchinenols A and B, Unprecedented Monocyclic Diterpenes with New Carbon Skeleton from the Aerial Parts of *Saururus chinensis*. Fitoterapia.

[33] Tran Q.T.N., Wong W.S.F., Chai C.L.L. (2017). Labdane Diterpenoids as Potential Anti-Inflammatory Agents. Pharmacol. Res..

[34] Peng Y., Zheng C., Wang Y.-N., Dai O. (2017). Novel Labdane Diterpenoids from the Aerial Parts of *Leonurus japonicus*. Phytochem. Lett..

[35] Rodríguez-Silverio J., Sánchez-Mendoza M.E., Rocha-González H.I., Reyes-García J.G., Flores-Murrieta F.J., López-Lorenzo Y., Quiñonez-Bastidas G.N., Arrieta J. (2021). Evaluation of the Antinociceptive, Antiallodynic, Antihyperalgesic and Anti-Inflammatory Effect of Polyalthic Acid. Molecules.

[36] Bozsó Z., Lapat V., Ott P.G., Móricz Á.M. (2024). Disparate Effects of Two Clerodane Diterpenes of Giant Goldenrod (*Solidago gigantea* Ait.) on *Bacillus spizizenii*. Int. J. Mol. Sci..

[37] Li R., Morris-Natschke S.L., Lee K.H. (2016). Clerodane Diterpenes: Sources, Structures, and Biological Activities. Nat. Prod. Rep..

[38] Martínez-Casares R.M., Hernández-Vázquez L., Mandujano A., Sánchez-Pérez L., Pérez-Gutiérrez S., Pérez-Ramos J. (2023). Anti-Inflammatory and Cytotoxic Activities of Clerodane-Type Diterpenes. Molecules.

[39] Reveglia P., Cimmino A., Masi M., Nocera P., Berova N., Ellestad G., Evidente A. (2018). Pimarane Diterpenes: Natural Source, Stereochemical Configuration, and Biological Activity. Chirality.

[40] Zhang Y., Xiong F., Zhang J.-J., Yue C.-F., Bi D.-W., Cheng B., Wu X.-W., Li Q., Zhang X.-J., Zhang R.-H. (2022). Euphzycopias A−I, Macrocyclic Diterpenes with NLRP3 Inflammasome Inhibitory Activity from *Euphorbia helioscopia* L.. Fitoterapia.

[41] González M.A. (2015). Aromatic Abietane Diterpenoids: Their Biological Activity and Synthesis. Nat. Prod. Rep..

[42] Bömke C., Tudzynski B. (2009). Diversity, Regulation, and Evolution of the Gibberellin Biosynthetic Pathway in Fungi Compared to Plants and Bacteria. Phytochemistry.

[43] Žiauka J., Kuusienė S. (2010). Different Inhibitors of the Gibberellin Biosynthesis Pathway Elicit Varied Responses during in Vitro Culture of Aspen (*Populus tremula* L.). Plant Cell Tissue Organ Cult. PCTOC.

[44] Wang M., Li H., Xu F., Gao X., Li J., Xu S., Zhang D., Wu X., Xu J., Hua H. (2018). Diterpenoid Lead Stevioside and Its Hydrolysis Products Steviol and Isosteviol: Biological Activity and Structural Modification. Eur. J. Med. Chem..

[45] Bu Q., Yang M., Yan X.-Y., Yao L.-G., Guo Y.-W., Liang L.-F. (2022). New Flexible Cembrane-Type Macrocyclic Diterpenes as TNF-*α* Inhibitors from the South China Sea Soft Coral *Sarcophyton mililatensis*. Int. J. Biol. Macromol..

[46] Athanasakoglou A., Kampranis S.C. (2019). Diatom Isoprenoids: Advances and Biotechnological Potential. Biotechnol. Adv..

[47] Frazão D.R., Cruz J.N., Santana de Oliveira M., Baia-da-Silva D.C., Nazário R.M.F., Rodrigues M.F.d.L., Saito M.T., Souza-Rodrigues R.D., Lima R.R. (2023). Evaluation of the Biological Activities of Copaiba (*Copaifera* spp.): A Comprehensive Review Based on Scientometric Analysis. Front. Pharmacol..

[48] Li H., Dickschat J.S. (2022). Diterpene Biosynthesis from Geranylgeranyl Diphosphate Analogues with Changed Reactivities Expands Skeletal Diversity. Angew. Chem. Int. Ed. Engl..

[49] Barbosa L.T.C., Vega M.R.G. (2017). Diterpenes from the Genus Xylopia. Rev. Virtual Quím..

[50] Peters R.J., Ravn M.M., Coates R.M., Croteau R.B. (2001). Bifunctional Abietadiene Synthase: Free Diffusive Transfer of the (+)-Copalyl Diphosphate Intermediate between Two Distinct Active Sites. J. Am. Chem. Soc..

[51] Ravn M.M., Peters R.J., Coates R.M., Croteau R. (2002). Mechanism of Abietadiene Synthase Catalysis: Stereochemistry and Stabilization of the Cryptic Pimarenyl Carbocation Intermediates. J. Am. Chem. Soc..

[52] Keeling C.I., Bohlmann J. (2006). Diterpene Resin Acids in Conifers. Phytochemistry.

[53] Hernández-Herrera A.D., Luna-Herrera J., Del Rocío González-Martínez M., Prieto-Hinojosa A.I., Turcios-Esquivel A.M., Castillo-Maldonado I., Delgadillo-Guzmán D., Ramírez-Moreno A., Bustos-Brito C., Esquivel B. (2023). Immunomodulatory Activity of Diterpenes over Innate Immunity and Cytokine Production in a Human Alveolar Epithelial Cell Line Infected with Mycobacterium Tuberculosis. Curr. Mol. Pharmacol..

[54] Mendes E., Ramalhete C., Duarte N. (2024). Myrsinane-Type Diterpenes: A Comprehensive Review on Structural Diversity, Chemistry and Biological Activities. Int. J. Mol. Sci..

[55] Wimmer K., Sachet M., Ramos C., Frantal S., Birnleitner H., Brostjan C., Exner R., Filipits M., Bago-Horvath Z., Rudas M. (2023). Differential Immunomodulatory Effects of Epirubicin/Cyclophosphamide and Docetaxel in Breast Cancer Patients. J. Exp. Clin. Cancer Res. CR.

[56] Hooda P., Malik R., Bhatia S., Al-Harrasi A., Najmi A., Zoghebi K., Halawi M.A., Makeen H.A., Mohan S. (2024). Phytoimmunomodulators: A Review of Natural Modulators for Complex Immune System. Heliyon.

[57] Díaz-Viciedo R., Hortelano S., Girón N., Massó J.M., Rodriguez B., Villar A., de las Heras B. (2008). Modulation of Inflammatory Responses by Diterpene Acids from *Helianthus annuus* L.. Biochem. Biophys. Res. Commun..

[58] Luo W., Bian X., Liu X., Zhang W., Xie Q., Feng L. (2023). A New Method for the Treatment of Myocardial Ischemia-Reperfusion Injury Based on γδT Cell-Mediated Immune Response. Front. Cardiovasc. Med..

[59] Habtemariam S. (2023). Anti-Inflammatory Therapeutic Mechanisms of Natural Products: Insight from Rosemary Diterpenes, Carnosic Acid and Carnosol. Biomedicines.

[60] Bardají D.K.R., da Silva J.J.M., Bianchi T.C., de Souza Eugênio D., de Oliveira P.F., Leandro L.F., Rogez H.L.G., Venezianni R.C.S., Ambrosio S.R., Tavares D.C. (2016). *Copaifera reticulata* Oleoresin: Chemical Characterization and Antibacterial Properties against Oral Pathogens. Anaerobe.

[61] Lemos M., Santin J.R., Mizuno C.S., Boeing T., de Sousa J.P.B., Nanayakkara D., Bastos J.K., de Andrade S.F. (2015). *Copaifera langsdorffii*: Evaluation of Potential Gastroprotective of Extract and Isolated Compounds Obtained from Leaves. Rev. Bras. Farmacogn..

[62] Li Y., Chi J., Zhang L., Wang F., Zhang W., Wang Z., Dai L. (2024). *Ent*-Kaurane Diterpenoids from *Isodon henryi* and Their Anti-Inflammatory Activities. Phytochemistry.

[63] Selener M.G., Borgo J., Sarratea M.B., Delfino M.A., Laurella L.C., Cerny N., Gomez J., Coll M., Malchiodi E.L., Bivona A.E. (2024). Trypanocidal and Anti-Inflammatory Effects of Three Ent-Kaurane Diterpenoids from *Gymnocoronis spilanthoides* Var. Subcordata (Asteraceae). Pharmaceutics.

[64] Símaro G.V., Lemos M., Mangabeira da Silva J.J., Ribeiro V.P., Arruda C., Schneider A.H., Wagner de Souza Wanderley C., Carneiro L.J., Mariano R.L., Ambrósio S.R. (2021). Antinociceptive and Anti-Inflammatory Activities of *Copaifera pubiflora* Benth Oleoresin and Its Major Metabolite Ent-Hardwickiic Acid. J. Ethnopharmacol..

[65] Pei X., Lou Y., Ren Q., Liu Y., Dai X., Ye M., Huang G., Cao J. (2024). Anti-Inflammatory Activities of Several Diterpenoids Isolated from *Hemionitis albofusca*. Naunyn. Schmiedebergs Arch. Pharmacol..

[66] Pei X., Zhang Z., Wang N., Huang G., Min X., Yang Y., Cao J. (2023). Onychiol B Attenuates Lipopolysaccharide-Induced Inflammation via MAPK/NF-κB Pathways and Acute Lung Injury in Vivo. Bioorganic Chem..

[67] Michavila Puente-Villegas S., Apaza Ticona L., Rumbero Sánchez Á., Acebes J.-L. (2024). Diterpenes of Pinus Pinaster Aiton with Anti-Inflammatory, Analgesic, and Antibacterial Activities. J. Ethnopharmacol..

[68] Kim E., Kang Y.-G., Kim Y.-J., Lee T.R., Yoo B.C., Jo M., Kim J.H., Kim J.-H., Kim D., Cho J.Y. (2019). Dehydroabietic Acid Suppresses Inflammatory Response Via Suppression of Src-, Syk-, and TAK1-Mediated Pathways. Int. J. Mol. Sci..

[69] Xu Y., Sun D., Xiong L., Zhang Z., Li Y., Liu K., Li H., Chen L. (2024). Phenolics and Terpenoids with Good Anti-Inflammatory Activity from the Fruits of *Amomum villosum* and the Anti-Inflammatory Mechanism of Active Diterpene. Bioorganic Chem..

[70] Zheng Y.-Y., Guo Z.-F., Chen H., Bao T.-R.-G., Gao X.-X., Wang A.-H., Jia J.-M. (2023). Diterpenoids from *Sigesbeckia glabrescens* with Anti-Inflammatory and AChE Inhibitory Activities. Phytochemistry.

[71] Wang Y., Sun D., Jiang Q., Xiong L., Zhang N., Pan Y., Li H., Chen L. (2023). Diterpenoids with Anti-Inflammatory Activity from *Euphorbia wallichii*. Phytochemistry.

[72] Grauso L., Falco B.d., Lucariello G., Capasso R., Lanzotti V. (2021). Diterpenes from *Euphorbia myrsinites* and Their Anti-Inflammatory Property. Planta Med..

[73] Leite P.M., Amorim J.M., Castilho R.O., Sangwan N.S., Farag M.A., Modolo L.V. (2022). Immunomodulatory Role of Terpenoids and Phytosteroids. Plants and Phytomolecules for Immunomodulation: Recent Trends and Advances.

[74] Sadeghi Z., Cerulli A., Marzocco S., Moridi Farimani M., Masullo M., Piacente S. (2023). Anti-Inflammatory Activity of Tanshinone-Related Diterpenes from *Perovskia artemisioides* Roots. J. Nat. Prod..

[75] Ngo T.M., Tran P.T., Hoang L.S., Lee J.-H., Min B.S., Kim J.A. (2021). Diterpenoids Isolated from the Root of Salvia Miltiorrhiza and Their Anti-Inflammatory Activity. Nat. Prod. Res..

[76] Vargas F.d.S., de Almeida P.D.O., Aranha E.S.P., Boleti A.P.d.A., Newton P., de Vasconcellos M.C., Junior V.F.V., Lima E.S. (2015). Biological Activities and Cytotoxicity of Diterpenes from *Copaifera* spp. Oleoresins. Molecules.

[77] Zhang B.-B., He B.-Q., Sun J.-B., Zeng B., Shi X.-J., Zhou Y., Niu Y., Nie S.-Q., Feng F., Liang Y. (2015). Diterpenoids from *Saliva plebeia* R. Br. and Their Antioxidant and Anti-Inflammatory Activities. Molecules.

[78] Liu Z.-G., Li Z.-L., Bai J., Meng D.-L., Li N., Pei Y.-H., Zhao F., Hua H.-M. (2014). Anti-Inflammatory Diterpenoids from the Roots of *Euphorbia ebracteolata*. J. Nat. Prod..

[79] Bulati M., Miceli V., Gallo A., Amico G., Carcione C., Pampalone M., Conaldi P.G. (2020). The Immunomodulatory Properties of the Human Amnion-Derived Mesenchymal Stromal/Stem Cells Are Induced by INF-γ Produced by Activated Lymphomonocytes and Are Mediated by Cell-To-Cell Contact and Soluble Factors. Front. Immunol..

[80] Senedese J.M., Rinaldi-Neto F., Furtado R.A., Nicollela H.D., de Souza L.D.R., Ribeiro A.B., Ferreira L.S., Magalhães G.M., Carlos I.Z., da Silva J.J.M. (2019). Chemopreventive Role of Copaifera Reticulata Ducke Oleoresin in Colon Carcinogenesis. Biomed. Pharmacother. Biomedecine Pharmacother..

[81] Karimdadi Sariani O., Eghbalpour S., Kazemi E., Rafiei Buzhani K., Zaker F. (2021). Pathogenic and Therapeutic Roles of Cytokines in Acute Myeloid Leukemia. Cytokine.

[82] Melgrati S., Sozzani S., Thelen M. (2023). Editorial: Insights in Cytokines and Soluble Mediators in Immunity: 2022. Front. Immunol..

[83] Gupta M., Chandan K., Sarwat M. (2022). Natural Products and Their Derivatives as Immune Check Point Inhibitors: Targeting Cytokine/Chemokine Signalling in Cancer. Semin. Cancer Biol..

[84] Arango Duque G., Descoteaux A. (2014). Macrophage Cytokines: Involvement in Immunity and Infectious Diseases. Front. Immunol..

[85] Lange A., Lange J., Jaskuła E. (2021). Cytokine Overproduction and Immune System Dysregulation in alloHSCT and COVID-19 Patients. Front. Immunol..

[86] Silva L.B., dos Santos Neto A.P., Maia S.M., dos Santos Guimarães C., Quidute I.L., Carvalho AD A., Júnior S.A., Leão J.C. (2019). The Role of TNF-α as a Proinflammatory Cytokine in Pathological Processes. Open Dent. J..

[87] Chhabra G., Singh C.K., Ndiaye M.A., Fedorowicz S., Molot A., Ahmad N. (2018). Prostate Cancer Chemoprevention by Natural Agents: Clinical Evidence and Potential Implications. Cancer Lett..

[88] Ri M.H., Ma J., Jin X. (2021). Development of Natural Products for Anti-PD-1/PD-L1 Immunotherapy against Cancer. J. Ethnopharmacol..

[89] Moudgil K.D., Venkatesha S.H. (2022). The Anti-Inflammatory and Immunomodulatory Activities of Natural Products to Control Autoimmune Inflammation. Int. J. Mol. Sci..

[90] Selmy A.H., Hegazy M.M., El-Hela A.A., Saleh A.M., El-Hamouly M.M. (2023). In Vitroand in Silico Studies of Neophytadiene; A Diterpene Isolated Fromaeschynomene Elaphroxylon (Guill. &Perr.) Taub. as Apoptotic Inducer. Egypt. J. Chem..

[91] Eggenhuizen P.J., Ng B.H., Ooi J.D. (2020). Treg Enhancing Therapies to Treat Autoimmune Diseases. Int. J. Mol. Sci..

[92] Ouyang W., Kolls J.K., Zheng Y. (2008). The Biological Functions of T Helper 17 Cell Effector Cytokines in Inflammation. Immunity.

[93] Wang Y., Jia L., Wu C.-Y. (2008). Triptolide Inhibits the Differentiation of Th17 Cells and Suppresses Collagen-Induced Arthritis. Scand. J. Immunol..

[94] Zhou J., Xiao C., Zhao L., Jia H., Zhao N., Lu C., Yang D., Tang J.C., Chan A.S.C., Lu A. (2006). The Effect of Triptolide on CD4^+^ and CD8^+^ Cells in Peyer’s Patch of SD Rats with Collagen Induced Arthritis. Int. Immunopharmacol..

[95] Xiao C., Lu C., Zhao L., Liu Z., Zhang W., He Y., Chen S., Tang J.C., Chan A.S., Lu A. (2006). The Effects of Triptolide on Enteric Mucosal Immune Responses of DBA/1 Mice with Collagen-Induced Arthritis. Planta Med..

[96] Zhao X., Ji W., Lu Y., Liu W., Guo F. (2023). Triptolide Regulates the Balance of Tfr/Tfh in Lupus Mice. Adv. Rheumatol..

[97] Zhao X., Tang X., Yan Q., Song H., Li Z., Wang D., Chen H., Sun L. (2019). Triptolide Ameliorates Lupus via the Induction of miR-125a-5p Mediating Treg Upregulation. Int. Immunopharmacol..

[98] Cerqueira F., Cordeiro-Da-Silva A., Gaspar-Marques C., Simões F., Pinto M.M.M., Nascimento M.S.J. (2004). Effect of Abietane Diterpenes from *Plectranthus grandidentatus* on T- and B-Lymphocyte Proliferation. Bioorg. Med. Chem..

[99] Ge Z.-P., Xu J.-B., Zhao P., Xiang M., Zhou Y., Lin Z.-M., Zuo J.-P., Zhao J.-X., Yue J.-M. (2024). Highly Modified Cephalotane-Type Diterpenoids from *Cephalotaxus fortunei* Var. Alpina and C. sinensis. Phytochemistry.

[100] Crossay E., Jullian V., Trinel M., Sagnat D., Hamel D., Groppi E., Rolland C., Stigliani J.-L., Mejia K., Cabanillas B.J. (2023). Daphnanes Diterpenes from the Latex of *Hura crepitans* L. and Their PKCζ-Dependent Anti-Proliferative Activity on Colorectal Cancer Cells. Bioorg. Med. Chem..

[101] Wei N., Li T., Chen H., Mei X., Cao B., Zhang Y. (2013). The Immunosuppressive Activity of Pseudolaric Acid B on T Lymphocytes. Phytother. Res..

[102] Muraguchi A., Miyazaki K., Kehrl J.H., Fauci A.S. (1984). Inhibition of Human B Cell Activation by Diterpine Forskolin: Interference with B Cell Growth Factor-Induced G1 to S Transition of the B Cell Cycle. J. Immunol. Baltim. Md 1950.

[103] Holte H., Torjesen P., Blomhoff H.K., Ruud E., Funderud S., Smeland E.B. (1988). Cyclic AMP Has the Ability to Influence Multiple Events during B Cell Stimulation. Eur. J. Immunol..

[104] Daďová P., Mikulová A., Jaroušek R., Chorvátová M., Uldrijan S., Kubala L. (2023). A Forskolin-Mediated Increase in cAMP Promotes T Helper Cell Differentiation into the Th1 and Th2 Subsets Rather than into the Th17 Subset. Int. Immunopharmacol..

[105] Rodriguez G., Ross J.A., Nagy Z.S., Kirken R.A. (2013). Forskolin-Inducible cAMP Pathway Negatively Regulates T-Cell Proliferation by Uncoupling the Interleukin-2 Receptor Complex. J. Biol. Chem..

[106] Dessauer C.W., Watts V.J., Ostrom R.S., Conti M., Dove S., Seifert R. (2017). International Union of Basic and Clinical Pharmacology. CI. Structures and Small Molecule Modulators of Mammalian Adenylyl Cyclases. Pharmacol. Rev..

[107] Waters L.R., Ahsan F.M., Wolf D.M., Shirihai O., Teitell M.A. (2018). Initial B Cell Activation Induces Metabolic Reprogramming and Mitochondrial Remodeling. iScience.

[108] Puri A., Saxena R., Saxena R.P., Saxena K.C., Srivastava V., Tandon J.S. (1993). Immunostimulant Agents from *Andrographis paniculata*. J. Nat. Prod..

[109] Ajaya Kumar R., Sridevi K., Vijaya Kumar N., Nanduri S., Rajagopal S. (2004). Anticancer and Immunostimulatory Compounds from *Andrographis paniculata*. J. Ethnopharmacol..

[110] Matsuda H., Morikawa T., Sakamoto Y., Toguchida I., Yoshikawa M. (2002). Labdane-Type Diterpenes with Inhibitory Effects on Increase in Vascular Permeability and Nitric Oxide Production from *Hedychium coronarium*. Bioorg. Med. Chem..

[111] Broz P., Dixit V.M. (2016). Inflammasomes: Mechanism of Assembly, Regulation and Signalling. Nat. Rev. Immunol..

[112] Strowig T., Henao-Mejia J., Elinav E., Flavell R. (2012). Inflammasomes in Health and Disease. Nature.

[113] Huang Z., Ye B., Han J., Kong F., Shan P., Lu Z., Huang Z., Huang W. (2018). NACHT, LRR and PYD Domains-Containing Protein 3 Inflammasome Is Activated and Inhibited by Berberine via Toll-like Receptor 4/Myeloid Differentiation Primary Response Gene 88/Nuclear Factor-κB Pathway, in Phorbol 12-Myristate 13-Acetate-Induced Macrophages. Mol. Med. Rep..

[114] Yang Y., Liu D., Cao H., Lu L., Zhang W., Liu C., Zeng Y., Shang F., Tao Y., Zhao B. (2024). Rosthornin B Alleviates Inflammatory Diseases via Directly Targeting NLRP3. FASEB J..

[115] Meng Z., Si C.-Y., Teng S., Yu X.-H., Li H.-Y. (2019). Tanshinone IIA Inhibits Lipopolysaccharide-induced Inflammatory Responses through the TLR4/TAK1/NF-κB Signaling Pathway in Vascular Smooth Muscle Cells. Int. J. Mol. Med..

[116] Dong X., Dong J., Zhang R., Fan L., Liu L., Wu G. (2009). Anti-Inflammatory Effects of Tanshinone IIA on Radiation-Induced Microglia BV-2 Cells Inflammatory Response. Cancer Biother. Radiopharm..

[117] Jin H., Peng X., He Y., Ruganzu J.B., Yang W. (2020). Tanshinone IIA Suppresses Lipopolysaccharide-Induced Neuroinflammatory Responses through NF-κB/MAPKs Signaling Pathways in Human U87 Astrocytoma Cells. Brain Res. Bull..

[118] Shang Q., Xu H., Huang L. (2012). Tanshinone IIA: A Promising Natural Cardioprotective Agent. Evid.-Based Complement. Altern. Med. ECAM.

[119] Su C.-C. (2016). Tanshinone IIA Decreases the Migratory Ability of AGS Cells by Decreasing the Protein Expression of Matrix Metalloproteinases, Nuclear Factor κB-P65 and Cyclooxygenase-2. Mol. Med. Rep..

[120] Li Y., Deng X., Zhuang W., Li Y., Xue H., Lv X., Zhu S. (2022). Tanshinone IIA Down-Regulates -Transforming Growth Factor Beta 1 to Relieve Renal Tubular Epithelial Cell Inflammation and Pyroptosis Caused by High Glucose. Bioengineered.

[121] Liu Q., Zhuang Y., Song X., Niu Q., Sun Q., Li X., Li N., Liu B., Huang F., Qiu Z. (2021). Tanshinone IIA Prevents LPS-Induced Inflammatory Responses in Mice via Inactivation of Succinate Dehydrogenase in Macrophages. Acta Pharmacol. Sin..

[122] Li D., Yang Z., Gao S., Zhang H., Fan G. (2022). Tanshinone IIA Ameliorates Myocardial Ischemia/Reperfusion Injury in Rats by Regulation of NLRP3 Inflammasome Activation and Th17 Cells Differentiation. Acta Cir. Bras..

[123] Aachoui Y., Chowdhury R.R., Fitch R.W., Ghosh S.K. (2011). Molecular Signatures of Phytol-Derived Immunostimulants in the Context of Chemokine-Cytokine Microenvironment and Enhanced Immune Response. Cell. Immunol..

[124] Pan X.-C., Liu Y., Cen Y.-Y., Xiong Y.-L., Li J.-M., Ding Y.-Y., Tong Y.-F., Liu T., Chen X.-H., Zhang H.-G. (2019). Dual Role of Triptolide in Interrupting the NLRP3 Inflammasome Pathway to Attenuate Cardiac Fibrosis. Int. J. Mol. Sci..

[125] Qian K., Zhang L., Shi K. (2019). Triptolide Prevents Osteoarthritis via Inhibiting Hsa-miR-20b. Inflammopharmacology.

[126] Li S., Sah D.K., Arjunan A., Ameer M.Y., Lee B., Jung Y.-D. (2025). Triptolide Suppresses IL-1β-Induced Expression of Interleukin-8 by Inhibiting ROS-Mediated ERK, AP-1, and NF-κB Molecules in Human Gastric Cancer AGS Cells. Front. Oncol..

[127] Guo W., Sun Y., Liu W., Wu X., Guo L., Cai P., Wu X., Wu X., Shen Y., Shu Y. (2014). Small Molecule-Driven Mitophagy-Mediated NLRP3 Inflammasome Inhibition Is Responsible for the Prevention of Colitis-Associated Cancer. Autophagy.

[128] Cabrera D., Wree A., Povero D., Solís N., Hernandez A., Pizarro M., Moshage H., Torres J., Feldstein A.E., Cabello-Verrugio C. (2017). Andrographolide Ameliorates Inflammation and Fibrogenesis and Attenuates Inflammasome Activation in Experimental Non-Alcoholic Steatohepatitis. Sci. Rep..

[129] Yu Y., Miao T., Xiao W., Mao B., Du L., Wang Y., Fu J. (2024). Andrographolide Attenuates NLRP3 Inflammasome Activation and Airway Inflammation in Exacerbation of Chronic Obstructive Pulmonary Disease. Drug Des. Devel. Ther..

[130] Das S., Mishra K.P., Ganju L., Singh S.B. (2017). Andrographolide—A Promising Therapeutic Agent, Negatively Regulates Glial Cell Derived Neurodegeneration of Prefrontal Cortex, Hippocampus and Working Memory Impairment. J. Neuroimmunol..

[131] Bao Z., Guan S., Cheng C., Wu S., Wong S.H., Kemeny D.M., Leung B.P., Wong W.S.F. (2009). A Novel Antiinflammatory Role for Andrographolide in Asthma via Inhibition of the Nuclear Factor-kappaB Pathway. Am. J. Respir. Crit. Care Med..

[132] Yao H., Zhao J., Zhu L., Xie Y., Zhao N., Yao R., Sun H., Han G. (2021). Protective Effect of the Effective Part of Andrographis Paniculata (Burm.f.) Nees on PM2.5-Induced Lung Injury in Rats by Modulating the NF-κB Pathway. J. Ethnopharmacol..

[133] Gao J., Cui J., Zhong H., Li Y., Liu W., Jiao C., Gao J., Jiang C., Guo W., Xu Q. (2020). Andrographolide Sulfonate Ameliorates Chronic Colitis Induced by TNBS in Mice via Decreasing Inflammation and Fibrosis. Int. Immunopharmacol..

[134] Kalergis A.M., Iruretagoyena M.I., Barrientos M.J., González P.A., Herrada A.A., Leiva E.D., Gutiérrez M.A., Riedel C.A., Bueno S.M., Jacobelli S.H. (2009). Modulation of Nuclear Factor-κB Activity Can Influence the Susceptibility to Systemic Lupus Erythematosus. Immunology.

[135] Li J., Huang L., He Z., Chen M., Ding Y., Yao Y., Duan Y., Zixuan L., Qi C., Zheng L. (2021). Andrographolide Suppresses the Growth and Metastasis of Luminal-Like Breast Cancer by Inhibiting the NF-κB/miR-21-5p/PDCD4 Signaling Pathway. Front. Cell Dev. Biol..

