# Correction: Almeida et al. Diterpenes: Nature’s Hidden Gems of Immunomodulation. *Int. J. Mol. Sci.* 2025, *26*, 2250

**DOI:** 10.3390/ijms27167483

**Published:** 2026-08-21

**Authors:** Josiane Elizabeth Almeida, André Correa de Oliveira, Carlos Eduardo de Castro Alves, Selino Monteiro Costa Filho, Elaine Cristina Pacheco de Oliveira, Juliana Pavan Zuliani, Gemilson Soares Pontes

**Affiliations:** 1 Graduate Program in Basic and Applied Immunology, Federal University of Amazonas (UFAM), Manaus 69080-900, AM, Brazil; josialmeida08@gmail.com; 2 Analytical Multidisciplinary Support Center, Federal University of Amazonas (UFAM), Manaus 69080-900, AM, Brazil; andrebiologo2011@gmail.com; 3 Laboratory of Virology and Immunology, Society, Environment and Health Coordination, National Institute of Amazonian Research (INPA), Manaus 69080-001, AM, Brazil; alveseduardo71@gmail.com; 4 Biotechnology and Medicinal Plants Laboratory, Federal University of Western Pará (UFOPA), Santarém 68040-255, PA, Brazil; selinofilho2018@gmail.com (S.M.C.F.); elaine.ibef@gmail.com (E.C.P.d.O.); 5 Laboratory of Cellular Immunology Applied to Health, Oswaldo Cruz Foundation (FIOCRUZ), Porto Velho 21040-900, RO, Brazil; juliana.zuliani@fiocruz.br; 6 Graduate Program in Hematological Sciences, University of Amazonas State (UEA), Manaus 69080-010, AM, Brazil

The following references, [11] and [13], have been retracted and should be removed from the original publication [1]:

11. Wu, W.; Li, Y.; Wu, X.; Liang, J.; You, W.; He, X.; Feng, Q.; Li, T.; Jia, X. Carnosic Acid Nanocluster-Based Framework Combined with PD-1 Inhibitors Impeded Tumorigenesis and Enhanced Immunotherapy in Hepatocellular Carcinoma. *Funct. Integr. Genom.* **2024**, *24*, 5.
13. Zhang, Q.-Q.; Ding, Y.; Lei, Y.; Qi, C.-L.; He, X.-D.; Lan, T.; Li, J.-C.; Gong, P.; Yang, X.; Geng, J.-G.; et al. Andrographolide Suppress Tumor Growth by Inhibiting TLR4/NF-κB Signaling Activation in Insulinoma. *Int. J. Biol. Sci.* **2014**, *10*, 404.

The scientific contents of [11] and [13] are replaced with the following publications:

11. Jantan, I.; Ahmad, W.; Bukhari, S.N.A. Plant-Derived Immunomodulators: An Insight on Their Preclinical Evaluation and Clinical Trials. *Front. Plant Sci.* **2015**, *6*, 655. https://doi.org/10.3389/fpls.2015.00655.
13. Ali Reza, A.S.M.; Nasrin, M.S.; Hossen, M.A.; Rahman, M.A.; Jantan, I.; Haque, M.A.; Sobarzo-Sánchez, E. Mechanistic Insight into Immunomodulatory Effects of Food-Functioned Plant Secondary Metabolites. *Crit. Rev. Food Sci. Nutr.* **2023**, *63*, 5546–5576. https://doi.org/10.1080/10408398.2021.2021138.

The authors state that the scientific conclusions are unaffected. This correction was approved by the Academic Editor. The original publication has also been updated.
